# The Lin28/let-7 axis is critical for myelination in the peripheral nervous system

**DOI:** 10.1038/ncomms9584

**Published:** 2015-10-14

**Authors:** Deniz Gökbuget, Jorge A. Pereira, Sven Bachofner, Antonin Marchais, Constance Ciaudo, Markus Stoffel, Johannes H. Schulte, Ueli Suter

**Affiliations:** 1 Department of Biology, Institute of Molecular Health Sciences, ETH Zurich, Otto-Stern-Weg 7, 8093 Zurich, Switzerland; 2 Department of Biology, Institute of Agricultural Sciences, ETH Zurich, Universitaetsstrasse 2, 8092 Zurich, Switzerland; 3 Department of Pediatric Oncology and Haematology, Children's Hospital Essen, 45122 Essen, Germany

## Abstract

MicroRNAs (miRNAs) are crucial regulators of myelination in the peripheral nervous system (PNS). However, the miRNAs species involved and the underlying mechanisms are largely unknown. We found that let-7 miRNAs are highly abundant during PNS myelination and that their levels are inversely correlated to the expression of lin28 homolog B (Lin28B), an antagonist of let-7 accumulation. Sustained expression of Lin28B and consequently reduced levels of let-7 miRNAs results in a failure of Schwann cell myelination in transgenic mouse models and in cell culture. Subsequent analyses revealed that let-7 miRNAs promote expression of the myelination-driving master transcription factor Krox20 (also known as Egr2) through suppression of myelination inhibitory Notch signalling. We conclude that the Lin28B/let-7 axis acts as a critical driver of PNS myelination, in particular by regulating myelination onset, identifying this pathway also as a potential therapeutic target in demyelinating diseases.

## Abstract (web summary)

MicroRNAs are known to regulate myelination in the peripheral nervous system, but the underlying mechanisms remain unclear. Here the authors show that the Lin28B/let-7 axis drives the onset of myelination by promoting Krox20 expression through suppression of Notch signalling.

## Graphical abstract

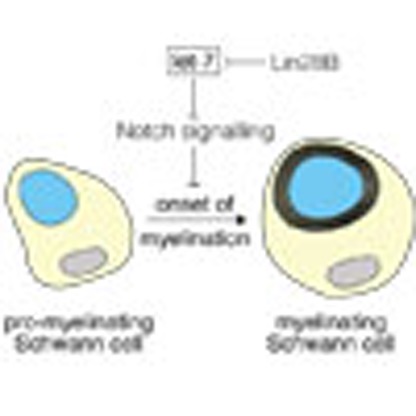

Posttranscriptional regulation by microRNAs (miRNAs) is ubiquitously important in cell differentiation and tumorigenesis^1^. Typically, the biogenesis of miRNAs involves sequential processing of the primary miRNA transcript by the RNAse III family enzymes Drosha and Dicer to yield a 22-nucleotide duplex. One strand of the mature miRNA duplex is loaded into the miRNA-induced silencing complex which targets mRNAs for translational repression and/or accelerated decay^2^.

The let-7 family comprises one of the evolutionary most conserved families of miRNAs, and multiple let-7 isoforms have crucial functions in development, homeostasis and tumour suppression^3^. Key regulators of let-7 expression are the RNA-binding proteins lin28 homolog A and B (Lin28A and Lin28B). Both block specifically let-7 biogenesis, and in turn, are targeted by let-7. Thus, the Lin28/let-7 system is able to act as a bi-stable switch that regulates the transition of opposing differentiation states with let-7 usually promoting this process and Lin28 opposing it^3^.

Myelination is a remarkable example of cell differentiation that ensures fast signal propagation in the vertebrate nervous system. The process is tightly controlled by the balance of negative and positive regulators, and in the PNS requires the integration of axonal and Schwann cell (SC)-derived signals^4^. Dicer-mediated miRNA biogenesis is indispensable for myelination in the PNS^5^,^6^,^7^,^8^. Dicer-deficient SCs arrest their development when they engage with axons in a 1:1 relationship, known as the pro-myelinating stage. Such mutant SCs fail to activate the correct myelination program and are unable to repress negative regulators of myelination, including Notch1 and Sox2. Several miRNA species were suggested as candidates that regulate myelination^9^,^10^. However, the physiologically relevant regulatory miRNA species involved have yet to be identified in this context.

Here we show a critical role for the Lin28B/let-7 pathway in the regulation of the onset of myelination. Developmental downregulation of Lin28B and consequently let-7 accumulation drives the onset of myelination by promoting Krox20 expression through suppression of Notch signalling.

## Results

### let-7 and Lin28B levels are anticorrelated upon myelination

**Figure 1:**
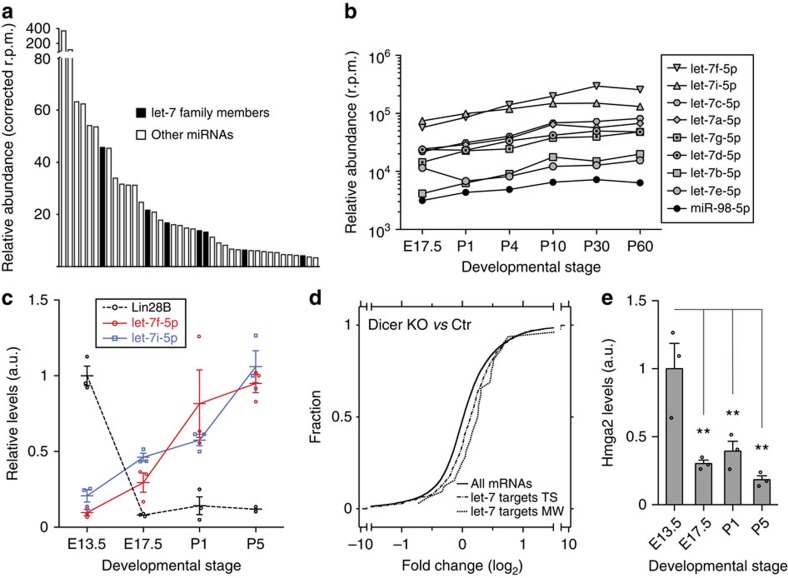
Let-7 miRNAs are highly expressed during myelination and inversely correlated to Lin28B expression. (**a**) Forty most abundant miRNAs in the SN of wild-type mice at PN4 corrected for values in SN of PN4 mice with SC-specific deletion of Dicer (Dicer KO; see the Methods section). (**b**) Mean levels of abundant let-7 isoforms and the Lin28-dependent miR-98-5p at embryonic day (ED) 17.5, PN1, PN4, PN10, PN30 and PN60 in SN of wild-type mice in reads per million (r.p.m.). (**c**) Levels of Lin28B mRNA and of mature let-7f-5p and let-7i-5p during SN development in wild-type mice, normalized to GAPDH mRNA for Lin28B and to snoRNA-202 for let-7 miRNAs (*n*=3 mice per time point). (**d**) Cumulative distribution of differential expression of all expressed mRNAs and let-7 targets, predicted with TargetScan (TS) or miRWalk (MW), in Dicer KO versus control SN at PN1. The rightward shifts in the curves for targets indicate enrichment compared with all expressed mRNAs. (**e**) Hmga2 mRNA levels during wild-type mouse SN development (*n*=3 mice per time point). Error bars: s.e.m. (**c**,**e**). One-way ANOVA with Dunnett's multiple comparison test **P<0.01 (**e**).

Given the importance of miRNAs during PNS myelination, we quantitatively assessed miRNA expression during sciatic nerve (SN) development by small RNA sequencing. We found that several members of the let-7 family are particularly strongly expressed in SCs during myelination (Fig. 1a,b; Supplementary Fig. 1a,b). Analysis at earlier developmental time points revealed that let-7 isoforms are induced prior to myelination onset, and that their levels are inversely correlated to those of Lin28B (Fig. 1c). Lin28A was not detectable at the time points examined (see the Methods section). Next, as a broad readout of let-7 function, we analysed differential expression of predicted let-7 targets in postnatal day (PN) 1 SN of mice lacking Dicer in SCs (Dicer KO) compared with control mice, using RNA sequencing. We found globally increased levels of let-7 targets in Dicer KO (Fig. 1d). In addition, analysis of developmental expression of Hmga2, a well-described let-7 target, revealed a prominent decline upon let-7 induction in SN (Fig. 1e). Consistently, Hmga2 protein levels were strongly elevated in SN of Dicer KO (Supplementary Fig. 1c,d). Taken together, our data demonstrate that the let-7 family is functionally enriched during PNS myelination and suggest that the decline of Lin28B expression before myelination might be causal in this context.

### Sustained Lin28B expression in SCs impairs myelination onset

**Figure 2:**
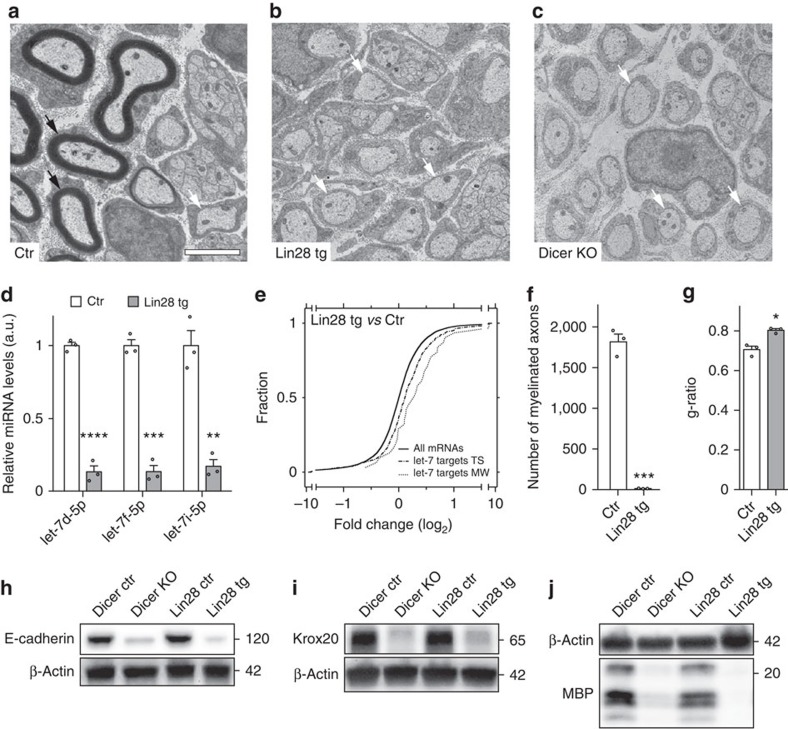
Sustained expression of Lin28B in SCs causes impaired onset of myelination. (**a**–**c**) Electron micrographs of SN of Ctr, Lin28 tg and Dicer KO at PN5. Examples of myelinated axons (black arrows) and 1:1 SC-axon relations (white arrows) are highlighted. (**d**) Levels of mature let-7f-5p, let-7i-5p and let-7d-5p in SN of Ctr and Lin28 tg at PN14, normalized to snoRNA-202 (*n*=3 mice per condition). (**e**) Cumulative distribution of differential expression of all expressed mRNAs and let-7 targets, predicted with TargetScan (TS) or miRWalk (MW), in Lin28 tg versus control SN at PN1. The rightward shifts in the curves for targets indicate enrichment compared with all expressed mRNAs. (**f**,**g**) Quantification of myelinated axons per cross-sections (**f**) and g-ratios (ratio of the inner axonal diameter to the total outer nerve fibre diameter) (**g**), determined on SN electron micrographs at PN5 (*n*=3 mice per condition). (**h**–**j**) Immunoblots of E-cadherin (**h**), Krox20 (**i**), MBP (**j**) and β-actin as loading control, from PN5 SN of Lin28 tg, Dicer KO and their respective controls. Numbers refer to estimated apparent molecular weights (kDa), except for MBP where the location of the 20 kDa size marker band is indicated. For quantifications, see Supplementary Fig. 3a–f. Scale bar, 2 μm (**a**–**c**). Error bars: s.e.m. Two-sided two-sample Student's *t*-test *P<0.05, **P<0.01, ***P<0.001, ****P<0.0001 (**d**,**f**,**g**).

To test if Lin28B repression and subsequently increased let-7 biogenesis is functionally required for PNS myelination, we employed conditionally inducible transgenic Lin28B mice^11^ and crossed them with Dhh-Cre mice^12^ to obtain animals with sustained SC-specific expression of Lin28B (Lin28 tg). Expression analysis confirmed that let-7 isoforms are strongly reduced in SN of these mice (Fig. 2d). Consequently, predicted let-7 targets were globally increased in Lin28 tg (Fig. 2e; Supplementary Fig. 1c,e), comparable to the observations in Dicer KO (Fig. 1d). Morphologically, at PN5, SN of Lin28 tg showed an almost complete lack of myelinated axons and were developmentally arrested at the pro-myelinating stage, comparable to PN5 SN with a Dicer KO background (Fig. 2a–c,f). Furthermore, the very few myelinated axons present in Lin28 tg had thinner myelin compared with control axons with the same calibre (Fig. 2g). At PN14, SN of Lin28 tg and Dicer KO still had drastic deficits with regard to the onset of myelination (Supplementary Fig. 2m–o). To determine whether the impairments due to sustained Lin28B expression were consistent in other PNS regions, we analysed dorsal (sensory nerve) and ventral (motor nerve) roots of Lin28 tg and Dicer KO mice (Supplementary Fig. 2). Morphological analysis revealed the same defective hallmarks as in SN, confirming the spatiotemporal consistency of the phenotype. To support the morphological observations at the molecular level, we examined expression of marker proteins associated with on-going myelination. Immunoblots of SN lysates from PN5 Lin28 tg and Dicer KO revealed strongly decreased levels of Krox20, E-cadherin and MBP compared with controls (Fig. 2h–j; Supplementary Fig. 3a–f). We conclude that sustained Lin28B expression leads to impaired onset of PNS myelination, pointing to a crucial regulatory role of the Lin28B/let-7 pathway in this process.

### Myelination and appropriate Krox20 expression require let-7

**Figure 3:**
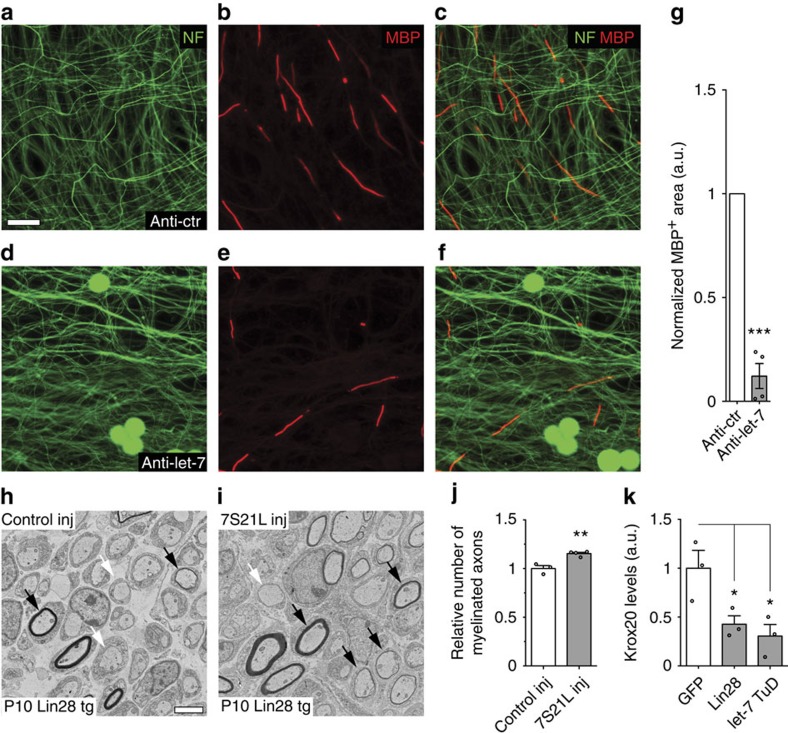
Lin28B-dependent let-7 miRNAs are necessary for myelination and appropriate Krox20 expression. (**a**–**f**) Immunostainings for neurofilament (NF; green) and MBP (red) of dissociated wild-type DRG explants treated with Anti-let-7 or control antagomir (Anti-ctr), prior to induction of myelination. (**g**) Quantification of MBP-positive area normalized to the NF-positive area (*n*=4 embryos). For individual embryonal cultures, Anti-ctr values were set to 1.(**h**,**i**) Electron micrographs of SN of Lin28 tg mice at PN10, injected with lentivirus expressing let-7S21L (7S21L inj) or control virus (Control inj) at PN3. Examples of myelinated axons (black arrows) and axons at the pro-myelinating stage (white arrows) are highlighted. (**j**) Quantification of the number of myelinated axons in let-7S21L (*n*=4 mice) or control injected (*n*=3 mice) SN, normalized to the number in the respective contralateral nerve. (**k**) Levels of Krox20 mRNA in primary SCs, induced for 1 h with Nrg1 48 h after transfection with Lin28, let-7 TuD or GFP-expressing vector. Primary SCs were grown and three randomly selected individual cultures per condition derived, individually processed and analysed. Two such sets of experiments were performed and the results of a representative one are shown. Scale bars equal 50 μm (**a**–**f**) and 3 μm (**h**,**i**); two-sided two-sample Student's *t*-test **P<0.01, ***P<0.001 (**g**,**j**). One-way ANOVA with Dunnett's multiple comparison test *P<0.05 (**k**). Error bars: s.e.m. (**g**,**j**,**k**).

Given the defects observed in response to sustained Lin28B overexpression, we asked if loss of let-7 miRNAs is the principal reason for the impaired myelination onset by targeting let-7 activity in myelinating *ex vivo* dorsal root ganglion cultures (DRG explants) using antagomirs^13^. Strongly diminished myelination was found in such anti-let-7-treated cultures (Fig. 3a–g) and similarly observed with DRG explants derived from Lin28 tg cultured under myelination-competent conditions (Supplementary Fig. 4a–i). *In vivo*, we sought to rescue the myelination deficits in Lin28 tg using the validated let-7S21L chimera that bypasses Lin28 binding and thus allows let-7 maturation despite Lin28 expression^14^. To achieve this, we generated lentiviral particles encoding let-7S21L followed by injection into PN3 Lin28 tg SN under conditions targeting SCs only (Supplementary Fig. 4j)^15^,^16^. After 7 days, the number of myelinated axons was significantly increased compared with control virus-injected Lin28 tg (Fig. 3h–j). Taken together, these results indicate that Lin28B-regulated let-7 isoforms are required for myelination.

Krox20 is the master regulator of PNS myelin gene expression and mice lacking Krox20 in SCs fail to start myelination, reminiscent of the Lin28 tg phenotype^17^. Since Krox20 was strongly reduced in Lin28 tg and Dicer KO, we investigated the role of Lin28B and let-7 in the regulation of neuregulin 1 (Nrg1)-dependent Krox20 expression. We transfected primary SCs with Lin28B or with let-7 targeting tough decoy expression vectors (let-7 TuD) prior to a 1 h Nrg1-stimulation^18^,^19^. Nrg1-induced Krox20 expression was decreased both in Lin28B and let-7 TuD-transfected cells (Fig. 3k) suggesting that Lin28B-dependent let-7 expression can affect Nrg1-induced Krox20 expression.

### The Notch pathway is hyperactive in Lin28 tg and Dicer KO

**Figure 4:**
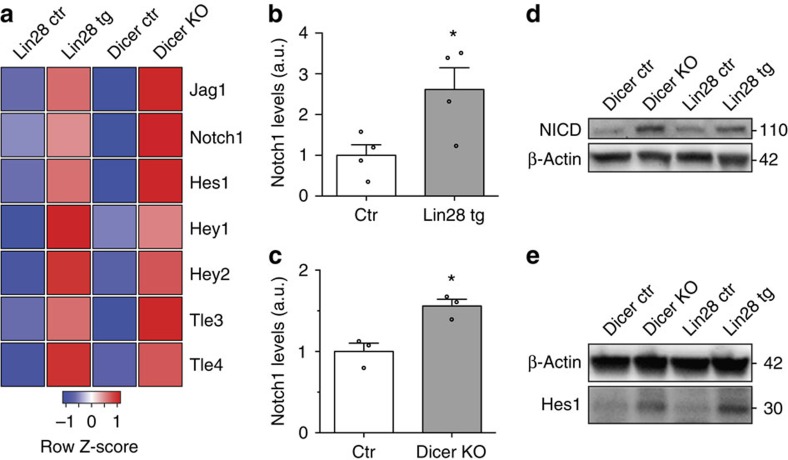
The myelination-inhibiting Notch pathway is hyperactive in Lin28 tg and Dicer KO. (**a**) Heatmap representation of significantly (*P*<0.05) regulated Notch pathway members in sequencing data from PN1 Lin28 tg and Dicer KO versus control SN (*n*=3 mice per condition). Colour intensities represent the Z-score for each row, red indicating higher expression and blue indicating lower expression. (**b**,**c**) Levels of Notch1 mRNA in SN of Lin28 tg and respective controls (*n*=4 mice per condition) (**b**) and in Dicer KO and respective controls (*n*=3 mice per condition) (**c**) at PN5. (**d**,**e**) Immunoblots of NICD (**d**), Hes1 (**e**), and β-actin as a loading control, from PN5 SN of Lin28 tg, Dicer KO and their respective controls. Numbers refer to estimated apparent molecular weights (kDa). For quantifications see Supplementary Fig. 3g–j. Error bars: s.e.m. Two-sided two-sample Student's *t*-test *P<0.05 (**b**,**c**).

To express Krox20 and to start myelination, SCs need to shift their balance of negative to positive regulators of myelination^4^. We hypothesized that let-7 isoforms might promote this shift by downregulating negative regulators of myelination. To obtain an unbiased collection of possible candidate targets, we compared PN1 SN of Lin28 tg, Dicer KO and controls by RNA sequencing. A MetaCore pathway analysis of transcripts that were upregulated in both mutants and carrying predicted let-7 sites in their 3′-UTRs, identified Notch signalling as the most-affected pathway. Thus, we focused on this pathway and found significantly increased RNA reads for Jag1, Notch1, Hes1, Hey1, Hey2, Tle3 and Tle4 in Lin28 tg and Dicer KO (Fig. 4a). Quantitative RT-PCR measurements confirmed increased Notch1 expression in both mutants (Fig. 4b,c). Next, we assessed Notch pathway activity in SN of Lin28 tg and Dicer KO by measuring protein levels of the intracellular domain of Notch1 (NICD) and its downstream target, Hes1. Both were increased compared with the controls (Fig. 4d,e; Supplementary Fig. 3g–j). These results identify let-7 as a critical regulator of the Notch pathway during myelination.

### let-7 promotes Krox20 and myelination via Notch1 suppression

**Figure 5:**
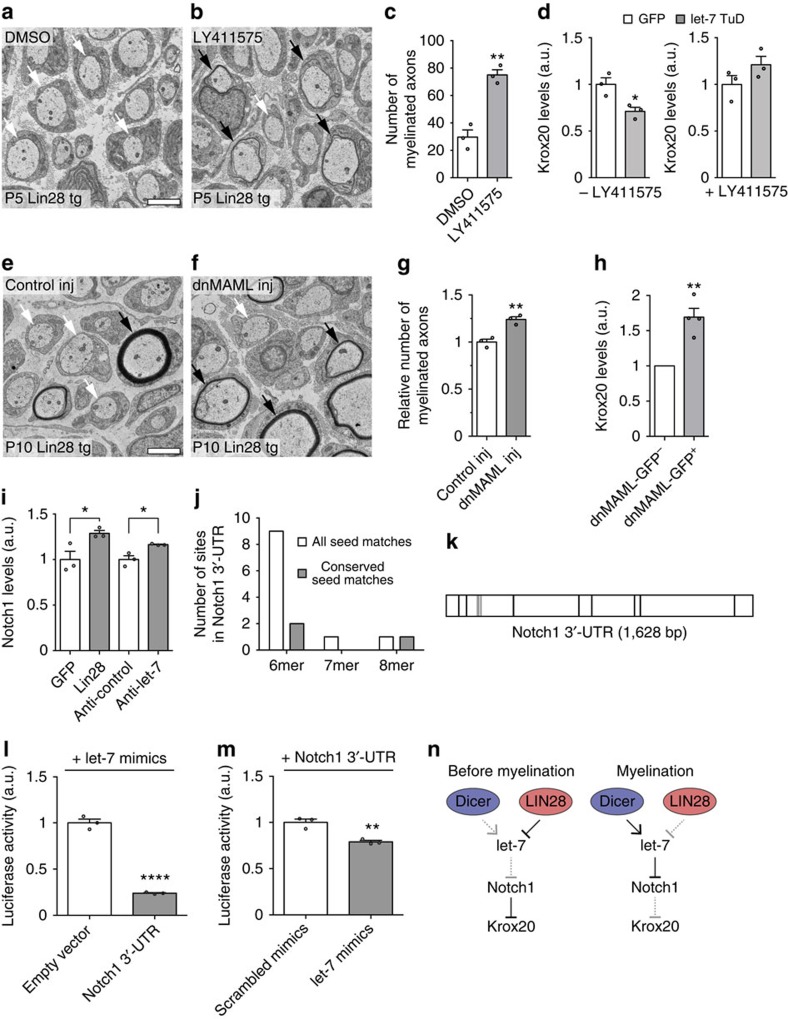
Notch1 suppression by let-7 promotes myelination through Krox20 expression. (**a**,**b**) Electron micrographs of PN5 Lin28 tg SN after injection of Notch signalling inhibitor LY411575 or DMSO solvent at both, PN3 and PN4. (**c**) Quantification of myelinated axons in LY411575- or DMSO-treated Lin28 tg per SN cross-section (*n*=3 mice per condition). (**d**) Krox20 mRNA levels in primary SCs transduced with GFP or let-7 TuD-expressing lentivirus prior to 1 h Nrg1 stimulation, with or without prior application of 10 μM LY411575. Primary SC were grown and three randomly selected individual cultures per condition derived, individually processed and analysed. (**e**,**f**) Electron micrographs of PN10 Lin28 tg SN, injected with lentivirus expressing dnMAML (dnMAML inj) or control virus (control inj) at PN3. (**g**) Quantification of myelinated axons in dnMAML- or control-injected SN, normalized to the respective contralateral nerve (*n*=3 mice per condition). (**h**) Krox20 mRNA levels in dnMAML-GFP-positive versus GFP^−^ cells, sorted 72 h after dnMAML lentivirus delivery to PN3 Lin28 tg SN (*n*=4 mice). (**i**) Notch1 mRNA levels 48 h after transfection of SpL201 cells with GFP or Lin28, or after application of control antagomir (Anti-control) or let-7f antagomir (Anti-let-7; *n*=3 cultures per condition). (**j**,**k**) Predicted let-7 seed interactions (allowing Wobble base pairs and one binding site per region) in Notch1 3′-UTR (**j**). Scheme of predicted seed interactions, conserved indicated in grey (**k**). RNAhybrid-predicted interactions are listed in Supplementary Data 1. (**l**,**m**) Luciferase activity in primary SCs, transfected 48 h before with let-7 mimics or scrambled mimics, and empty pmirGLO or pmirGLO harbouring the Notch1 3′-UTR. Primary SCs were grown and three randomly selected individual cultures per condition derived, individually processed and analysed. Three such sets of experiments were performed and the results of a representative one are shown. (**n**) Model summarizing the role of the Lin28B/let-7 axis in myelination. Solid lines (active mechanisms), dotted lines (inactive mechanisms). Myelinated axons (black arrows), pro-myelinating stage axons (white arrows), scale bars, 2 μm (**a**,**b**,**e**,**f**). Error bars: s.e.m., two-sided two-sample Student's *t*-test *P<0.05, **P<0.01, ***P<0.001, ****P<0.0001 (**c**,**d**,**g**,**h**,**i**,**l**,**m**).

Notch1 is a well-known negative regulator of myelination. Sustained Notch1 expression interferes with PNS myelination by opposing the forward driver Krox20 (ref. 20). Thus, we further investigated the interplay between let-7 and the Notch pathway. To test if aberrantly increased Notch signalling interferes with myelination in Lin28 tg, we aimed at rescuing myelination in Lin28 tg with the Notch signalling inhibitor LY411575, a γ-secretase blocker. Consistent with our hypothesis, treatment with LY411575 caused a prominent increase in the number of myelinated axons (Fig. 5a–c). Furthermore, LY411575 treatment prevented the decrease of Krox20 expression in primary SCs expressing let-7-TuD (Fig. 5d). In addition, delivery of lentiviral particles expressing dominant-negative mastermind-like protein (dnMAML)^21^, inhibitory to Notch signalling, into PN3 Lin28 tg SN led to an increased number of myelinated axons (Fig. 5e–g). This increase was linked to elevated Krox20 levels in dnMAML-infected cells sorted from dnMAML-injected Lin28 tg SN (Fig. 5h). To examine if Notch1 levels are dependent on let-7, we analysed Notch1 expression in differentiated cells of the SC precursor line SpL201, 48 h after transfection with Lin28 or treatment with anti-let-7 antagomirs. Both conditions led to increased Notch1 levels compared with controls (Fig. 5i). These combined results concord with our *in vivo* observations, and suggest that let-7 isoforms positively affect Krox20 expression by downregulating Notch1 expression. To address if this regulation occurs directly through let-7 binding, we searched for predicted let-7 seed matches in the Notch1 3′-UTR. *In silico* analysis revealed multiple conserved seed matches (Fig. 5j,k; Supplementary Data 1). To biochemically validate these seed matches as *bona fide* let-7 target sites, the Notch1 3′-UTR was cloned into a luciferase reporter construct and luciferase activity was assayed in the presence or absence of let-7 mimics after transfection of primary SCs. Let-7 mimics caused decreased luciferase activity compared with scrambled controls or the empty vector (Fig. 5l,m). We conclude that let-7 isoforms promote Krox20 expression at least partly via targeting the Notch1 3′-UTR and suppression of Notch signalling in SCs (Fig. 5n).

## Discussion

In our studies, we have discovered a physiologically critical function of the Lin28/let-7 pathway during terminal cell differentiation. First, we identified let-7 isoforms as a major class of regulatory miRNAs that promote PNS myelination. Through targeting and reducing Notch signalling, let-7 miRNAs inhibit a central pathway opposing myelination. Second, we found that Lin28B is a negative regulator of PNS myelination. Since Lin28B repression is necessary to enable let-7 biogenesis, we propose a model in which Lin28B repression and concomitant let-7 induction drives myelination through inhibition of the Notch pathway.

Our data show that Lin28B is developmentally repressed prior to the onset of myelination and that its sustained expression impairs the timely onset of myelination. The decline in Lin28B levels and concomitant induction of let-7 accumulation are in agreement with findings in other cellular systems showing that let-7 miRNAs are associated with more differentiated states, while Lin28 supports the maintenance of undifferentiated states^3^. Lin28B is itself a target of let-7 and this may partially explain the reduction of Lin28B expression during early SC development. Furthermore, Sox2 is an important regulator of glial lineage identity and is repressed prior to myelination^22^,^23^. Since Sox2 promotes Lin28 expression in embryonic stem cells (ESCs) and neural progenitors^24^,^25^, Sox2 reduction might also contribute to the reduced Lin28B expression during SC lineage progression. In ESCs, Lin28 is part of the Sox2-Nanog-Oct4-Tcf3 network, which maintains ESCs in their pluripotent state while priming them for rapid let-7-mediated differentiation^25^. Whether a related concept applies to the regulation of nerve-associated SC precursors that can still give rise to multiple non-SC types, including skin melanocytes, odontoblasts and parasympathetic neurons, remains to be elucidated^26^,^27^,^28^,^29^.

Sustained Lin28B expression caused a major arrest of the axon-SC unit at the pro-myelinating stage. The severity of the phenotype indicates a function of Lin28B as a negative regulator of PNS myelination, consistent with established criteria^30^. These include expression before myelination and inactivation upon myelination. Furthermore, Lin28B opposes signals promoting myelination by negatively affecting let-7-mediated Notch pathway repression.

With the identification of the promoting function of let-7 in myelination, we follow up on findings demonstrating that global loss of Dicer-mediated miRNA biogenesis in SCs impairs myelination, in particular myelination onset^5^,^6^,^7^,^8^. The similarities between Lin28 tg and Dicer KO support the contribution of common regulatory mechanisms to this defect. Our data indicate that these mechanisms involve the loss of let-7 isoforms and the consequent ectopic expression of their targets. Furthermore, we have shown that let-7 miRNAs are necessary for myelination in DRG explants and that myelination deficits in Lin28 tg can be improved by Lin28-resistant let-7. Nevertheless, we do not exclude a contribution of let-7-independent functions of Lin28B in its role as a negative regulator of myelination. Additional analyses using inducible systems to alter Dicer or Lin28 expression, specifically at later developmental stages and in the adult, will shed further light on this issue and the potential role of other miRNA regulatory pathways. In fact, the defect of Lin28 tg and Dicer KO at the onset of myelination precludes the detailed analysis of possible regulatory functions of miRNAs at later stages of myelination and in myelin maintenance. Our expression analysis revealed, however, several miRNA species besides let-7 isoforms that are abundant at different stages and thus provides a valuable resource for future studies aimed at the investigation of additional physiological miRNA functions in myelin biology.

Our studies revealed that targeting and downregulation of Notch signalling by let-7 contributes to the onset of myelination, based on the fact that Notch1 is known to prevent the timely onset of myelination by opposing Krox20 activation. Given the involvement of Notch signalling in many other aspects of metazoan development and disease, these findings warrant further studies of the relationship between Notch signalling and the antagonizing role of let-7 in other systems. Likewise, functions of the Lin28/let-7 regulatory axis during remyelination, after injury^31^ or in demyelinating diseases, also merit further investigations, particularly in the prospect of exploring novel therapeutic approaches for demyelinating diseases.

## Methods

### Animals

Mice (*Mus musculus*) heterozygous for the *Lin28b* transgene were crossed with transgenic mice expressing Cre recombinase under *dhh* regulatory elements (*Dhh*^*Cre*^) to obtain mice expressing Lin28B specifically in SCs. Ablation of Dicer was obtained by crossing homozygous *Dicer1*^loxP/loxP^(ref. 32) with *Dhh*^*Cre*^ mice heterozygous for the *Dicer1*^loxP/loxP^ allele. Animals from both genders were used throughout experiments at the ages of embryonic day (ED)13.5, ED17.5, PN1, PN4, PN5, PN6, PN10, PN14, PN30 and PN60. Lin28 tg mice were backcrossed for at least 6 generations, *Dicer1*^loxP/loxP^ mice for at least 7 generations and *Dhh*^*Cre*^ mice for >10 generations, to C57BL6. Transgenic animals were identified by genomic PCR. Animal use was approved by the veterinary office of the Canton of Zurich.

### Cell culture

Primary rat SCs were obtained from PN2 to PN3 rat SN as described and grown in DMEM containing 10% FBS (Life Technologies), 1:500 penicillin/streptomycin (Life Technologies), 4 μg ml^−1^ bovine pituitary extract (Life Technologies) and 2 μM Forskolin (Sigma) at 37 °C/5% CO_2_ on cell culture dishes coated with poly-D-lysine (Sigma)^33^. Differentiation was induced after 18 h incubation in DMEM containing 1 × N2 supplement (Life Technologies) by addition of 25 ng ml^−1^ Nrg1 (human heregulin beta1, Peprotech). When dissociated cultures were used >98% of them were SCs.

Mycoplasm-free SpL201 cells were grown in DMEM containing 10% FBS and 10 ng ml^−1^ EGF (Peprotech)^34^. For differentiation, medium was changed to DMEM containing 10% FBS and 20 μM Forskolin.

### DRG explant cultures

DRGs from ED13.5 embryos were prepared^35^. In brief, DRGs were extracted and dissociated. Cultures were seeded at a density of 3 DRGs per well and grown in NB media for 11 days. To induce myelination, NB media was exchanged by C media containing 50 μg ml^−1^ ascorbic acid and cultures were kept in that media for another 7 days. Antagomirs were added 3 days before induction of myelination.

### Constructs and nucleotides

To generate pmirGLO-Notch1-UTR, the 3′-UTR of Notch1 was amplified by PCR from genomic DNA and ligated into pmirGLO (Promega). For expression of let-7-TuD, the let-7f-TuD-forward and let-7f-TuD-reverse DNA oligonucleotides (Supplementary Table 1) were annealed and cloned into pSicoR-Δ3'-loxP (modified version of pSicoR with deleted 3'-loxP site). Lin28 expression vector was acquired from Addgene (Addgene plasmid 26358)ref. 36.

Scrambled control and let-7f antagomir (Supplementary Table 1) were cholesterol-conjugated and had the same terminal phosphothioate and 2-*O*-methyl modifications as described in Krutzfeldt *et al*.^13^, and were used at a final concentration of 5 μg ml^−1^. As mimics, hsa-let-7f-5p and control miRIDIAN mimics (Thermo) were used at a final concentration of 10 nM.

### Deep sequencing

For small RNA sequencing, RNA from SN of ED17.5, PN1, PN4, PN10, PN30 and PN60 C57BL6 animals (two biological samples from independent mice for each time point) was extracted with Qiazol (Qiagen) according to the manufacturer's instructions. In addition, RNA from rat SCs (one culture) differentiated for 56 h with dibutyryl-cAMP (Sigma) and from PN4 Dicer KO (two biological samples from independent mice) was included to account for which miRNAs are SC-derived and which are not. Library preparation and sequencing was performed with Fasteris SA. Briefly, after RNA quality assessment with a Bioanalyzer 2100 (Agilent), the fraction of 15–35 nt was purified using gel electrophoresis and linked to Illumina TruSeq adapters. For multiplexing, barcodes were introduced during PCR amplification. Sequencing was performed using the Illumina HiSeq 2000 platform with a read length of 50 bp. The ncPRO pipeline was used to map the reads against the mouse genome (version mm9) and the rat genome (version rn4) ref. 37. We used Bowtie with default options (but -m 5000 and -e 50). The miRNA quantities were normalized by the number of mapped reads from the corresponding library and then multiplied by 1,000,000 to obtain the unit in reads per million (r.p.m.). The R software package DESeq R (Version 1.14.0) (ref. 38) was used to assess the statistical significance of differences in gene expression. Genes showing altered expression with adjusted *P*-value of <0.05 (Benjamini and Hochberg method) were considered as differentially expressed. Complete sequencing data are deposited in NCBI GEO database (GSE64562).

For RNA sequencing, total RNA from SN of PN1 Lin28 tg, Dicer KO and controls (three mice per condition, analysed individually) was extracted using Qiazol (Qiagen) according to the manufacturer's instructions. Library preparation, sequencing and mapping of reads was performed at the Functional Genomics Centre in Zurich (FGCZ). The quality of isolated RNA was determined with a Bioanalyzer 2100 (Agilent). Only those samples with a 260 nm/280 nm ratio between 1.8–2.1 and a 28S/18S ratio within 1.5–2 were further processed. The TruSeq RNA Sample Prep Kit v2 (Illumina) was used in the succeeding steps. Briefly, total RNA samples (100–1,000 ng) were depleted of ribosomal RNA using Ribo Zero Gold (Epicentre) and then reverse transcribed into double-stranded cDNA. The cDNA samples were fragmented, end-repaired and polyadenylated before ligation of TruSeq adapters containing the index for multiplexing fragments containing TruSeq adapters on both ends were selectively enriched with PCR. The quality and quantity of the enriched libraries were validated using Qubit (1.0) Fluorometer and the Caliper GX LabChip GX (Caliper Life Sciences). The product is a smear with an average fragment size of ∼260 bp. The libraries were normalized to 10 nM in Tris-HCl 10 mM (pH 8.5) with 0.1% Tween 20. The TruSeq PE Cluster Kit v3-cBot-HS or TruSeq SR Cluster Kit v3-cBot-HS (Illumina) was used for cluster generation using 10 pM of pooled normalized libraries on the cBOT. Sequencing was performed on the Illumina HiSeq 2000 paired end at 2 × 101 bp using the TruSeq SBS Kit v3-HS (Illumina). *In silico*, the obtained raw reads were first cleaned by removing adapter sequences and trimming the low-quality ends. Sequence alignment of the resulting high-quality reads to the mouse reference genome (build mm10) and quantification of gene level expression was carried out using RSEM (version 1.2.12) (ref. 39). The R software package from Bioconductor, DESeq2 (version 1.4.5) (ref. 38), was used to assess the statistical significance of differences in gene expression. Genes showing altered expression with adjusted *P*-value<0.05 (Benjamini and Hochberg method) were considered as differentially expressed. Complete sequencing data are deposited in NCBI GEO database (GSE64562).

### Electron microscopy

Mice were killed with a single injection of Pentobarbital (150 mg kg^−1^ intraperitoneally). Tissue was subsequently fixed with 3% glutardialdehyde and 4% paraformaldehyde in 0.1 M phosphate buffer. The tissue was further treated with 2% osmium tetroxide (EMS), dehydrated over a series of acetone gradients and embedded in Spurrs resin (EMS). Semithin sections were stained with a 1% toluidine blue solution for light microscope analysis. Ultrathin sections (65 nm) were imaged with a FEI Morgagni 268 TEM for high-resolution micrographs. Additional sections (99 nm) were used to perform morphological quantifications. The sections were collected on ITO coverslips (Optics Balzers) and the entire surface of the nerve section was imaged and analysed with either a Zeiss Gemini Leo 1530 FEG or Zeiss Merlin FEG scanning electron microscopes attached to ATLAS modules (Zeiss), which allows imaging of large areas at high resolution.

### Flow cytometry

Mice infected with a lentiviral vector expressing dnMAML, a GFP fusion protein, were killed 72 h post virus injection. Briefly, after removal of the perineurium, SN were digested for 70 min in 4 mg ml^−1^ collagenase (Sigma), 1.2 mg ml^−1^ hyaluronidase (Sigma) and 0.3 mg ml^−1^ trypsin inhibitor (Sigma) in 25 mM HEPES in MEM (Life Technologies). GFP^+^ and GFP^−^ cells were sorted using a FACSAria III (BD Biosciences).

### Generation of lentivirus and *in vivo* injections

Lentivirus was produced by transfecting HEK293T cells with each lentiviral construct together with the packaging vectors psPAX2 and pMD2.G using Lipofectamine 2000 (Life Technologies) according to the instructions of the manufacturer. The supernatant was collected 72 h after transfection and frozen in liquid nitrogen. Concentration of lentivirus and delivery of lentivirus to the SN was performed as previously described^40^. Treatment with the Notch signalling (γ-secretase) inhibitor LY411575 (Sigma) was performed through injections beneath the *gluteus superficialis* and *biceps femoris* muscles. Animals were injected on 2 consecutive days at PN3 and PN4 with 0.335 mg kg^−1^ per day of LY411575 diluted in a mixture of 10% DMSO and 45% 2-hydroxypropyl-β-cyclodextrin in PBS.

### Immunoblots

After removal of the perineurium, SNs were homogenized in microcentrifugation tubes (Eppendorf) using chilled pestles (Argos Technologies) in lysis buffer (2% SDS, 10 mM NaCl, 25 mM Tris-HCl pH 7.4, proteinase inhibitor cocktail (Sigma), phosphatase inhibitor (Roche)). Lysates were supplemented with sample buffer (6.2 mM Tris, 1% β-mercaptoethanol, 2% glycerol) and further processed using standard SDS-PAGE and western blot methods. The following antibodies were used: Notch1 (MAB5352, Millipore, 1:1,000), HES1 (ab71559, Abcam, 1:1,000), EGR2 (13491-1-AP, Proteintech, 1:1,000), E-cadherin (ALX-804-202-C100, Enzo, 1:1,000), HMGA2 (#8179, Cell Signalling, 1:1,000), β-actin (A5316, Sigma, 1:5,000), MBP (MCA409s, Serotec, 1:1,000). Secondary antibodies were obtained from Jackson and Promega. Signal detection was carried out using Fusion FX7 (Vilber Lourmat) and band intensities were quantified using Quantity One software (Biorad). Uncropped blots are shown in Supplementary Fig. 5. Size markers refer to All Blue Precision Protein Standards (Biorad).

### Immunofluorescence

Cells and explant cultures were fixed with 4% PFA for 10 min and with methanol at −20 °C for 20 min. After washing with PBS, samples were incubated in 5% NGS (Life Technologies) and 0.2% Triton-X-100 in PBS (blocking buffer) for 1 h. Primary and secondary antibodies were diluted in blocking buffer and incubated overnight (primary antibodies) and for 1 h (secondary antibodies). As primary antibodies, mouse-anti Neurofilament 160 (N5264, 1:200, Sigma) and rat-anti MBP (MCA409s, Serotec, 1:200) were used. Secondary antibodies were Alexa-Fluor-Dye-coupled, obtained from Life Technologies. Nuclei were stained by incubation with 0.1 ng ml^−1^ DAPI (Sigma) in PBS for 10 min.

### Image analysis

For quantifications of myelination of DRG explants, 10 random images were taken at × 10 magnification for each condition and the area of MBP and NF immunoreactivities was analysed using the CellProfiler software. Briefly, for each channel the background was subtracted resulting in binary images. At first the illumination was separately corrected for all images of each channel. Large objects like neuron cell bodies were excluded during this step. For each condition, the ratio of MBP-positive area divided by NF-positive area was calculated.

### *In silico* resources

Seed matches of let-7 were predicted using miRWalk, TargetScan and RNAhybrid^41^,^42^,^43^. Level of conservation was manually checked for each seed match using the conservation track in the UCSC genome browser. Pathway analysis was performed using Metacore (Thomson Reuters).

### Luciferase assays

Luciferase assays were performed with the Dual-Luciferase Reporter Assay System (Promega) according to the manufacturer's instructions. Primary rat SCs were transfected with miRNA mimics, pmirGLO (Promega) or pmirGLO-Notch1-3-UTR 48 h before the assay.

### Reverse transcription and real-time PCR

Total RNA was extracted from homogenized SN previously separated from the perineurium, or cells, using Qiazol (Qiagen). For Taqman miRNA assays, 2–5 ng total RNA was used for reverse transcription. For total cDNA, 50 to 100 ng total RNA was reverse transcribed using the Maxima First Strand cDNA Synthesis Kit (Thermo). Products of reverse transcription were diluted 1:10 in ddH_2_O and 2 μl was used for real-time PCR using 2 × FastStart Essential DNA Green Master Mix (Roche). Analysis was carried out with at least three technical replicates per sample. Primer sequences can be found in Supplementary Table 1. If no amplicons appeared before 40 cycles, the expression was judged as not detectable.

### Statistics statement

Statistical analysis was performed using Graphpad Prism 6 or Microsoft Excel. The two-sided two-sample Student's *t*-test (assuming equal variances) was chosen to determine statistical significance between two groups and was applied unless stated otherwise. Due to the small sample sizes (*n*<5 for most *t*-tests), assumptions of how well normality and equal variances fit the data could not be reliably assessed. Non-parametric tests such as the Wilcoxon rank-sum test were not used because of too low power. For data sets with values very close to zero in one of the compared groups, statistical significance was confirmed after natural logarithmic transformation: Fig. 2f (*P*<0.0001), Fig. 3g (*P*=0.036), Supplementary Fig. 1d (*P*<0.0001), Supplementary Fig. 1e (*P*<0.0001), Supplementary Fig. 3e (*P*<0.0001), Supplementary Fig. 3f (*P*<0.0001), Supplementary Fig. 4i (*P*=0.009). Detailed information about error bars and sample sizes are included in all figure legends and further specifications can be found in the Methods section. Sample size was not predetermined by statistical methods. Randomization was not applied unless stated otherwise. The investigators were not blinded to group allocation during the experiments because mutant animals display obvious behavioural and morphological impairments. No samples or data were excluded from the analysis.

## Additional information

**Accession codes:** Complete sequencing data are deposited in NCBI GEO database (GSE64562).

**How to cite this article:** Gökbuget, D. *et al*. The Lin28/let-7 axis is critical for myelination in the peripheral nervous system. *Nat. Commun.* 6:8584 doi: 10.1038/ncomms9584 (2015).

## Supplementary Material

Supplementary Information Supplementary Figures 1–5 and Supplementary Table 1

Supplementary Data 1 Predicted interactions of let-7 with the Notch1 3'-UTR using RNAhybrid.
