# Supplementary material for: The Lin28/let-7 axis is critical for myelination in the peripheral nervous system
Source: Nat Commun. 2015 Oct 14;6:8584. doi: 10.1038/ncomms9584 (PMC4634210; doi:10.1038/ncomms9584)
Supplement: Supplementary Information — Supplementary Figures 1–5 and Supplementary Table 1 [file ncomms9584-s1.pdf]

Supplementary Information

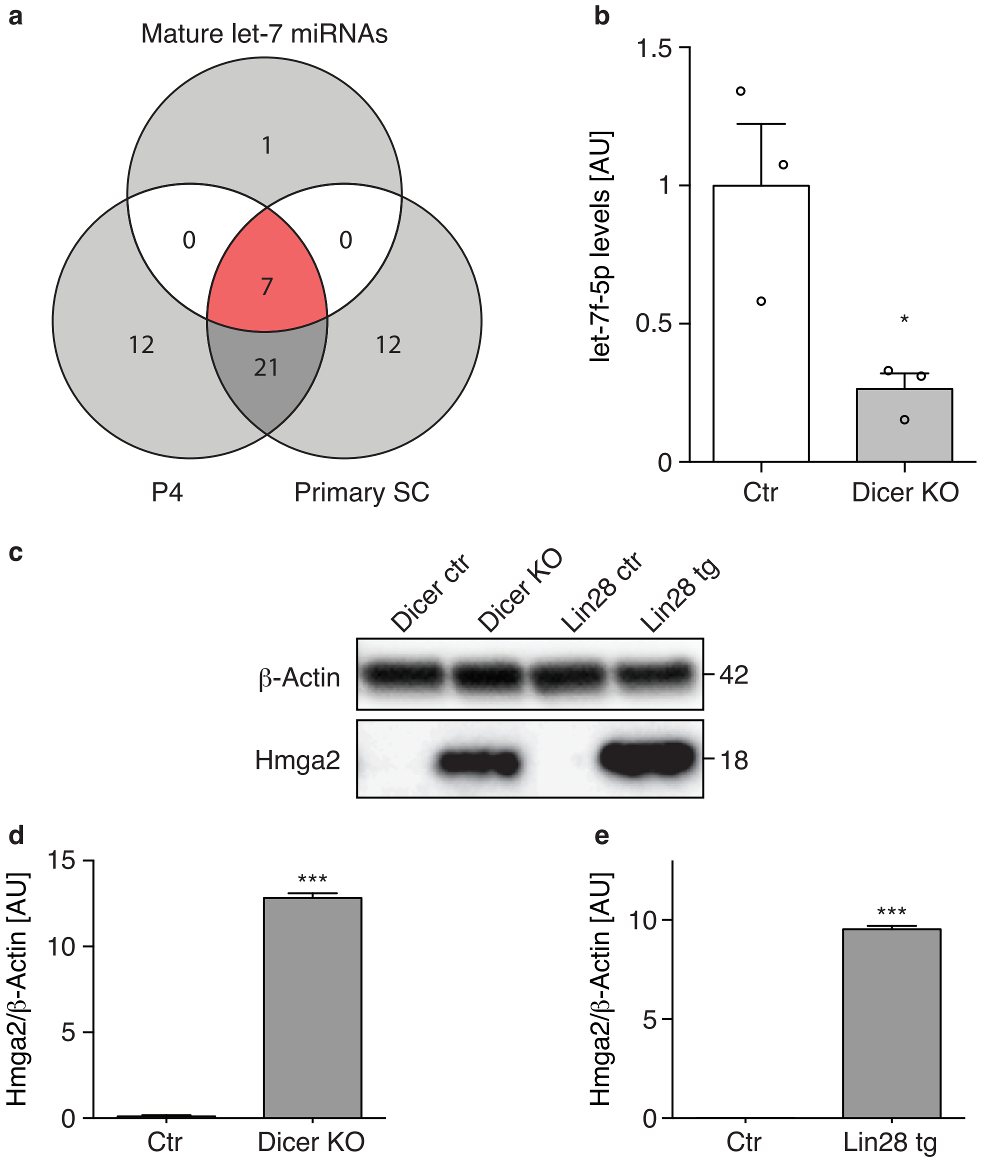

Supplementary Figure 1. Small RNA sequencing of cultured differentiated SCs and Hmga2 protein expression analysis. (**a**) Venn diagram showing the common presence of most let-7 family members (annotated in mm9) among the 40 most enriched miRNAs in P4 wild-type (corrected for P4 Dicer expressed miRNAs) and in cultured differentiated SCs. (**b**) Levels of mature let-7f-5p in P4 Dicer KO and controls, normalized to snoRNA-202 (n = 3 mice per condition). (**c**) Immunoblot of Hmga2 from P5 SN of Dicer KO, Lin28 tg and their respective controls. β-Actin serves as loading control. Numbers refer to estimated apparent molecular weights [kDa]. (**d,e**) Quantification of protein levels of Hmga2 in P5 Dicer KO and Lin28 tg normalized to β-Actin levels (n = 6 mice per condition). Error bars: s.e.m., two-sided two-sample Student's t-test $^{*}P < 0.05$, $^{**}P < 0.01$, $^{***}P < 0.001$ (**b,d,e**).

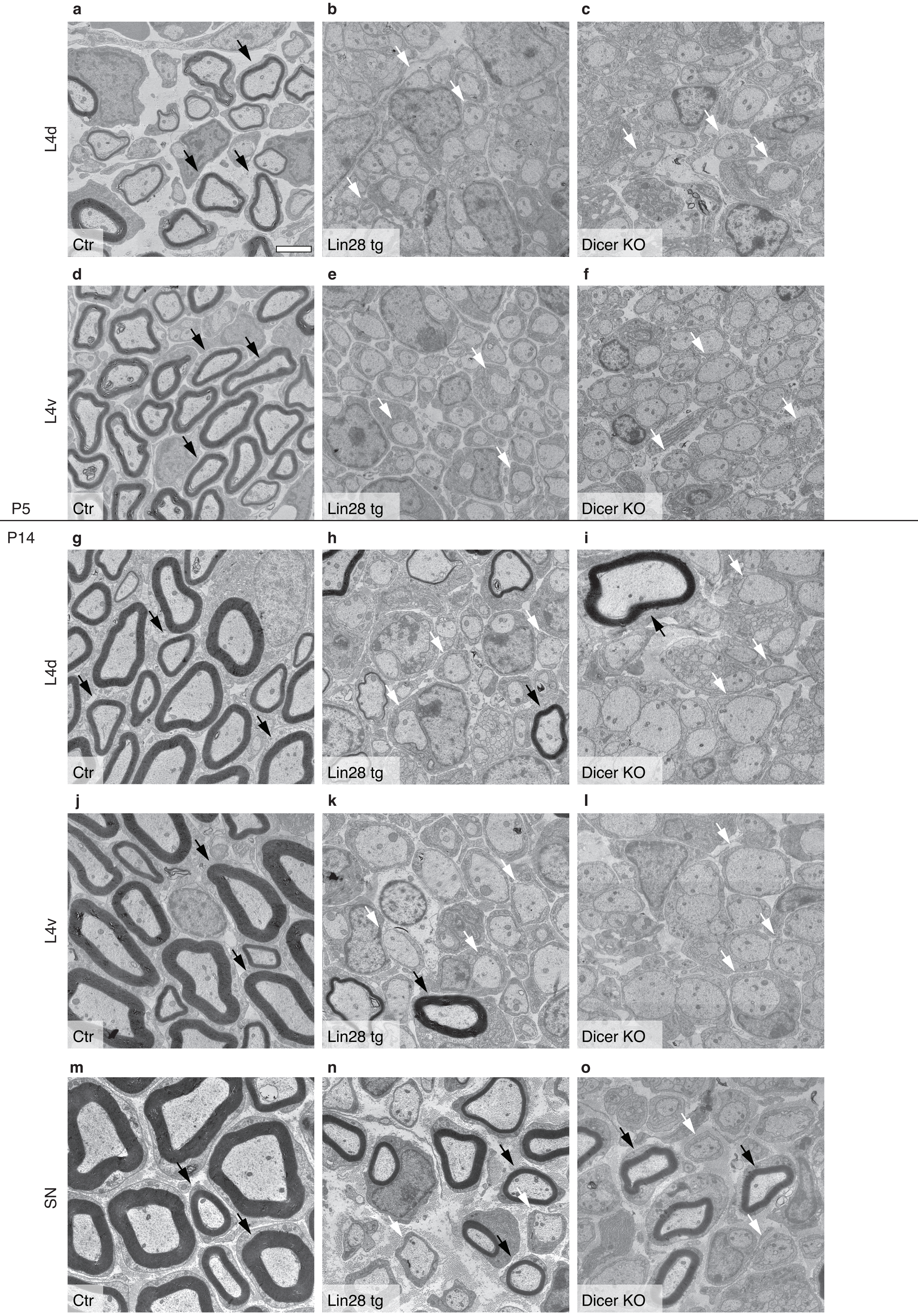
a
b
c
L4d
Ctr
Lin28 tg
Dicer KO
d
e
f
L4v
Ctr
Lin28 tg
Dicer KO
P5
P14
g
h
i
L4d
Ctr
Lin28 tg
Dicer KO
j
k
l
L4v
Ctr
Lin28 tg
Dicer KO
m
n
o
SN
Ctr
Lin28 tg
Dicer KO

Supplementary Figure 2. Morphological analysis of Lin28 tg at P5 and P14. (**a-f**) Electron micrographs of Lin28 tg, Dicer KO and controls from dorsal (L4d, **a-c**) and ventral (L4v, **d-f**) lumbar 4 roots at P5. (**g-o**) Electron micrographs of Lin28 tg, Dicer KO and controls at P14 from L4d (**g-i**), L4v (**j-l**) and SN (**m-o**); Myelinated axons (black arrows) and axons at the pro-myelinating stage (white arrows) are highlighted. Images are representatives from at least 3 mice per condition. Scale bar = 2 μm (**a-o**).

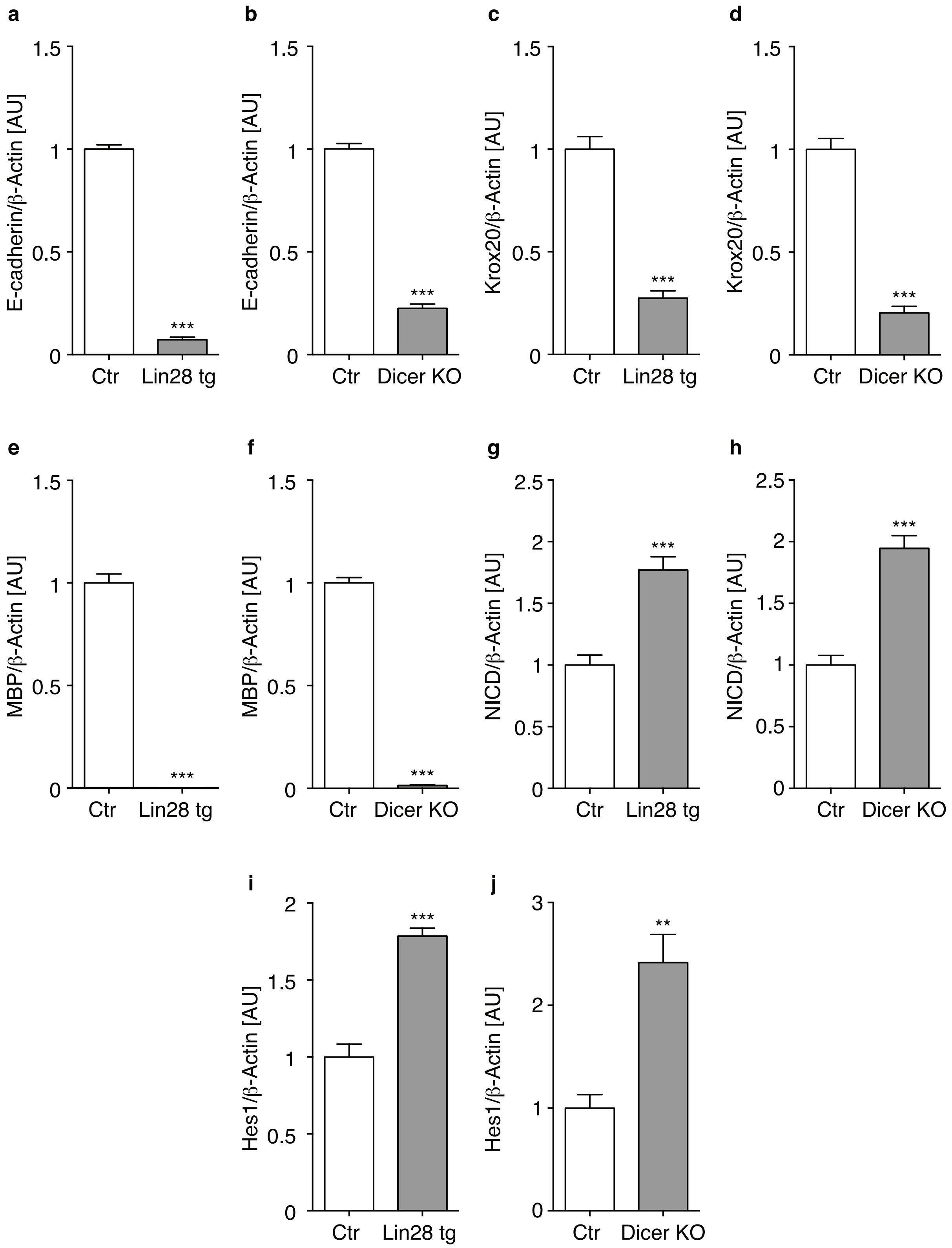

Supplementary Figure 3. Quantification of immunoblots. (**a-j**) Quantification of protein levels of E-cadherin in P5 Lin28 tg and Dicer KO (**a,b**), Krox20 in P5 Lin28 tg and Dicer KO (**c,d**), MBP in P5 Lin28 tg and Dicer KO (**e,f**), NICD in P5 Lin28 tg and Dicer KO (**g,h**) and Hes1 in P5 Lin28 tg and Dicer KO (**i,j**), with their respective controls. Levels were normalized to β-Actin levels. Six (**a-i**) and nine (**j**) mice were analysed for each condition. Error bars: s.e.m. Two-sided two-sample Student's t-test $*P < 0.05$, $**P < 0.01$, $***P < 0.001$ (**a-j**).

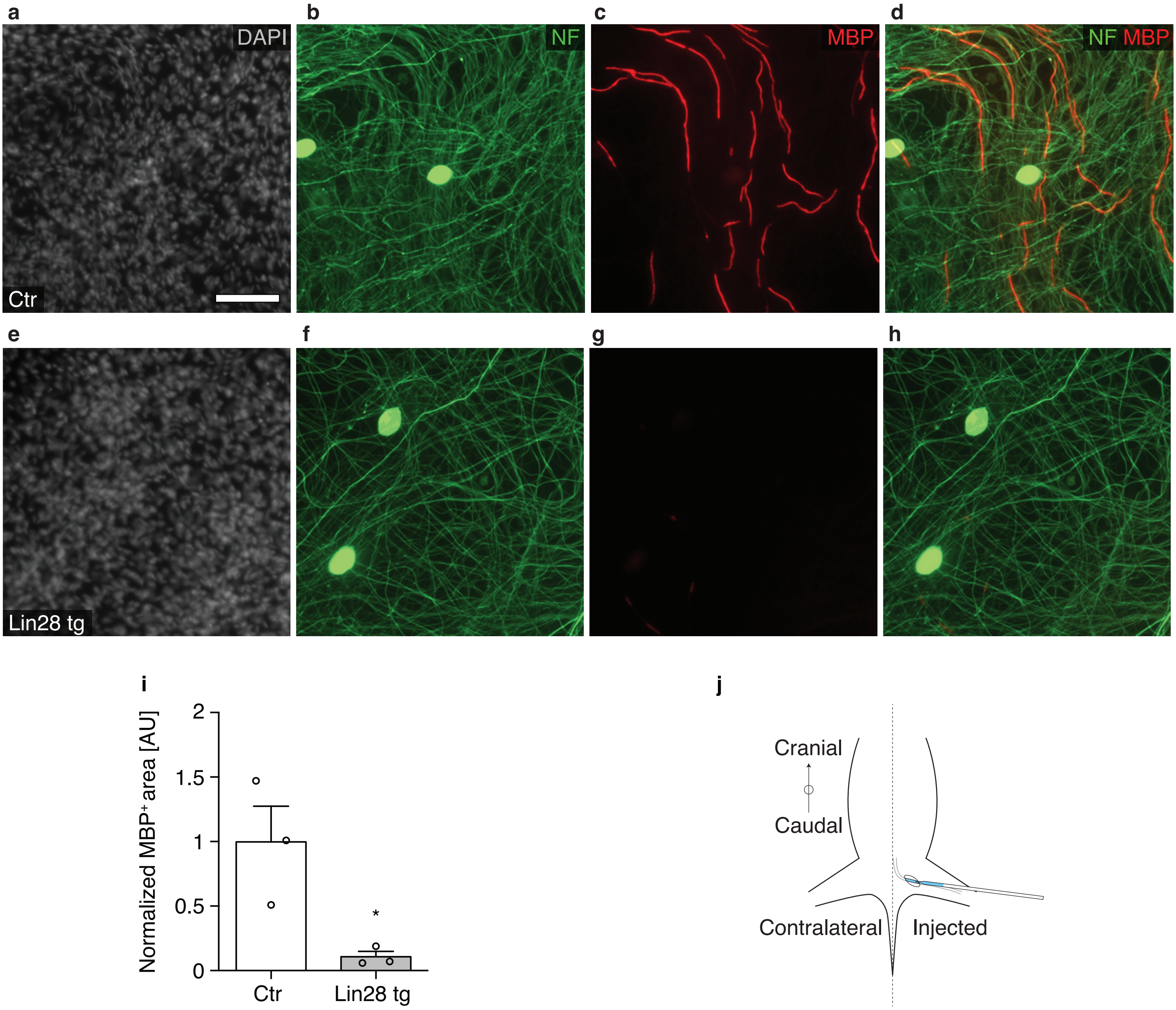

Supplementary Figure 4. Myelination is suppressed in Lin28 tg DRG explant cultures. (**a-h**) Dissociated DRG explants from Lin28 tg and controls immunostained for NF (green) and MBP (red). Nuclei were stained with DAPI (blue, false coloured in white). (**i**) Quantification of MBP^+ area in Lin28 tg and controls (Ctr) after normalization to NF^+ area (n = 3 embryos per condition). (**j**) Scheme of injection of lentivirus into SN; Scale bar = 100 μm (**a-h**); Error bars: s.e.m. two-sided two-sample Student's t-test $^*P < 0.05$ (**i**).

Fig. 2h

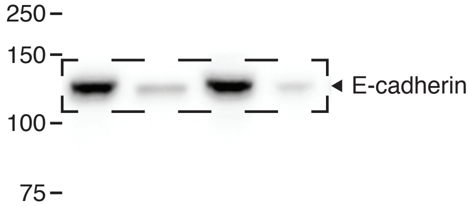

Fig. 2h

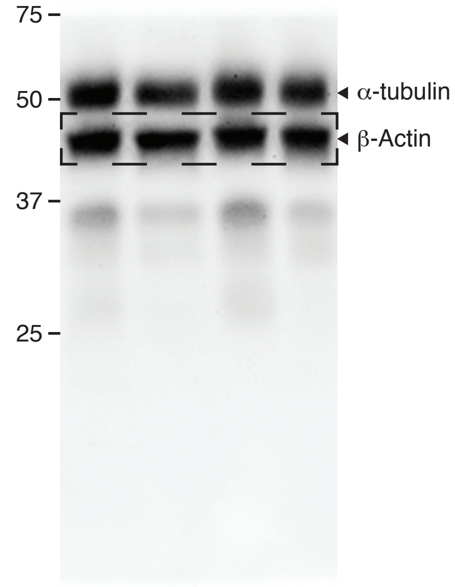

Fig. 2j

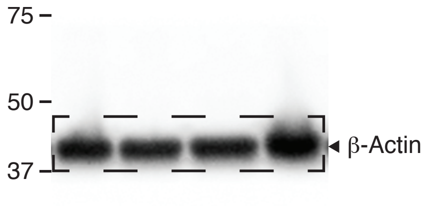

Fig. 2j

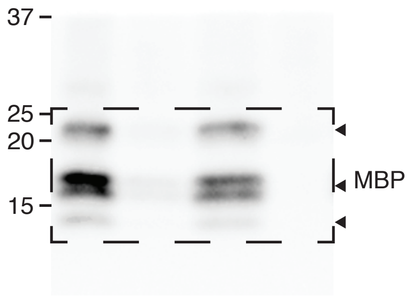

Fig. 2i

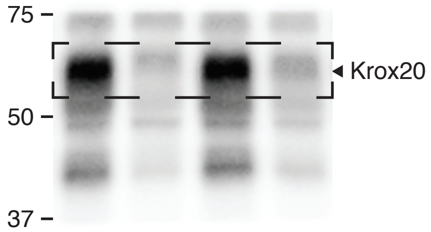

reprobed

Fig. 2i

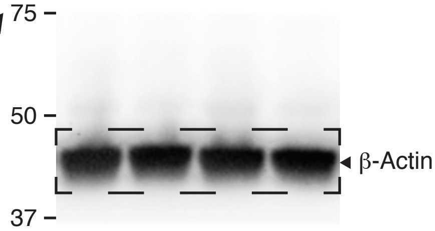

Fig. 4d

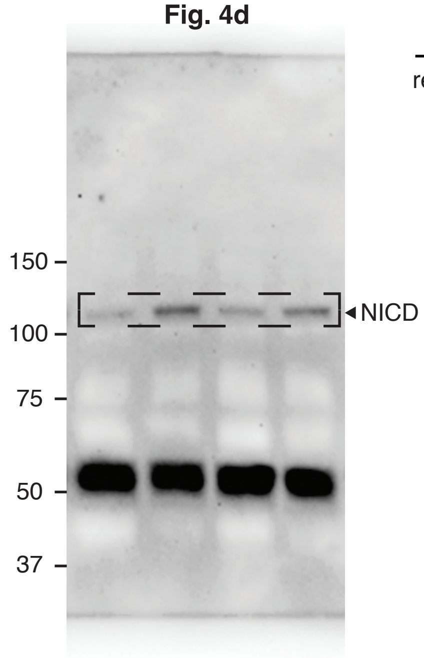

reprobed

Fig. 4d

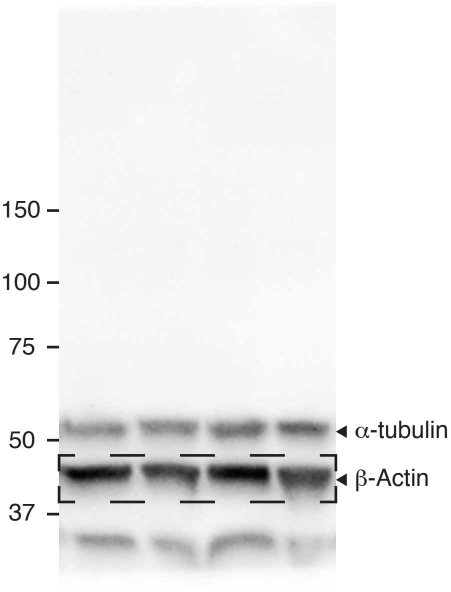

Fig. 4e

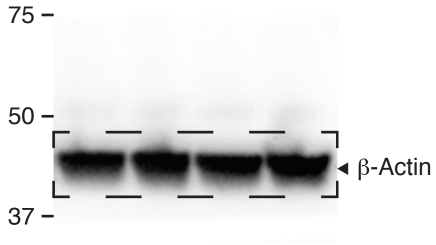

Fig. 4e

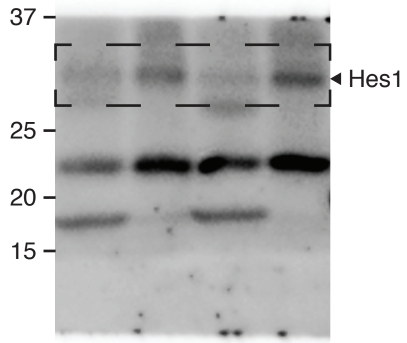

Supplementary Fig. 1c

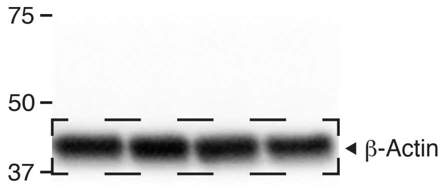

Supplementary Fig. 1c

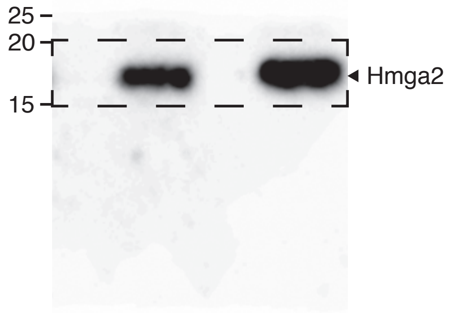

Supplementary Figure 5. Uncropped immunoblots displayed in manuscript. Cropped and quantified regions are highlighted (dashed boxes). After transfer, membranes were cut as follows: Fig. 2h (2 pieces, cut at 75 kDa). Fig. 2j (3 pieces, cut at 37 kDa and 75 kDa). Fig. 2i (3 pieces, cut at 37 kDa and 75 kDa). Fig. 4d (2 pieces, cut at 30 kDa). Fig. 4e (3 pieces, cut at 37 kDa and 75 kDa). Supplementary Fig. 1c (4 pieces, cut at 25 kDa, 37 kDa and 75 kDa). Pieces not shown were used for unrelated probing. SDS polyacrylamide gels were used as 4-15% gradient gels (Fig. 2j, Supplementary Fig. 1c) and 10% gels (Figs. 2h, 2i, 4d, 4e). Numbers refer to molecular weight size markers [kDa].

Supplementary Table 1. List of nucleotides.

Name	Species	Sequence (5'-3')
GAPDH_F	mouse	GGTGAAGGTCGGTGTGAACGGATTTGG
GAPDH_R	mouse	GGTCAATGAAGGGGTCGTTGATGGCAAC
Lin28A_qPCR_F	mouse	GGCATCTGTAAGTGGTTCAACG
Lin28A_qPCR_R	mouse	GCCAGTGACACGGATGGATT
Lin28B_qPCR_F	mouse	AACGTGCGCATGGGATTCGG
Lin28B_qPCR_R	mouse	TGACTCAAGGCCTTTGGGGGAT
Hmga2_qPCR_F	mouse	CAGCAAAAACAAGAGCCCCTC
Hmga2_qPCR_R	mouse	CGACTTGTTGTGGCCATTTCC
Hes1_qPCR_F	rat & mouse	CTACCCCAGCCAGTGTCAACACGAC
Hes1_qPCR_R	rat & mouse	AGCTTGGAATGCCGGGAGCTATCTTTC
Krox20_qPCR_F	rat & mouse	ACAGCCTCTACCCGGTGGAAGAC
Krox20_qPCR_R	rat & mouse	CAGAGATGGGAGCGAAGCTACTCGGATA
Notch1_qPCR_F	rat & mouse	TCCCACAGGCTGGCAAGGTCAAAC
Notch1_qPCR_R	rat & mouse	AGCGGTAGCTGCCATTGGTGTTCTG
Notch1_UTR_F	mouse	TTATTTCAGTGCTGGGTGGCCC
Notch1_UTR_R	mouse	CGTCCCAGATCACCCACATTCC
let7f_TuD_F	mouse	TGACGGCGCTAGGATCATCAACAACTATACAATCATCTT ACTACCTCACAAGTATTCTGGTCACAGAATACAACAACT ATACAATCATCTTACTACCTCACAAGATGATCCTAGCGC CGTCTTTTTTC
let7f_TuD_R	mouse	TCGAGAAAAAAGACGGCGCTAGGATCATCTTGTGAGGT AGTAAGATGATTGTATAGTTGTTGTATTCTGTGACCAGA ATACTTGTGAGGTAGTAAGATGATTGTATAGTTGTTGAT GATCCTAGCGCCGTCA
anti-let-7	mouse	AACUAUACAAUCUACUACCUCA
anti-Ctr	mouse	UCAACAUCAGUCUGAUAAGCUA
