# Supplementary material for: The Lin28/let-7 axis is critical for myelination in the peripheral nervous system
Source: Nat Commun. 2015 Oct 14;6:8584. doi: 10.1038/ncomms9584 (PMC4634210; doi:10.1038/ncomms9584)
Supplement: Supplementary Data 1 — Predicted interactions of let-7 with the Notch1 3'-UTR using RNAhybrid. [file ncomms9584-s2.pdf]

Supplementary Data 1. Predicted interactions of let-7 with the Notch1 3'-UTR using RNAhybrid.

dataset: 1
TARGET : mm_Notch1_3UTR
length: 1628
MIRNA : mmu-let-7f-5p
length: 22

mfe: -21.5 kcal/mol
p-value: undefined

position 766
```
target 5' A   C     GG  G  G      C 3'
           AGC AUGCA  GU UG UGCCUC
           UUG UAUGU  UA AU AUGGAG
miRNA  3'     A         G  G      U 5'
```

dataset: 1
TARGET : mm_Notch1_3UTR
length: 1628
MIRNA : mmu-let-7f-5p
length: 22

mfe: -17.8 kcal/mol
p-value: undefined

position 163
```
target 5'   A     UUUU    UCCACAGAAAC        U 3'
             UGUAC    AUUU           ACUGCCUU
             AUAUG    UAGA           UGAUGGAG
miRNA  3' UUG     U                          U 5'
```

dataset: 1
TARGET : mm_Notch1_3UTR
length: 1628
MIRNA : mmu-let-7f-5p
length: 22

mfe: -17.1 kcal/mol
p-value: undefined

position 345
```
target 5' U     UU       UGAU      U 3'
           GCUGU   AUUUAU    UGCUUC
           UGAUA   UAGAUG    AUGGAG
miRNA  3' U     UGU                U 5'
```

dataset: 1
TARGET : mm_Notch1_3UTR
length: 1628
MIRNA : mmu-let-7f-5p
length: 22

mfe: -17.0 kcal/mol
p-value: undefined

position 43
```
target 5'  C    UG  AGUCCUU  C G       G 3'
            CUGU  GG       UC A UGCUUCA
            GAUA  UU       AG U AUGGAGU
miRNA  3' UU    UG           A G         5'
```

dataset: 1
TARGET : mm_Notch1_3UTR
length: 1628
MIRNA : mmu-let-7f-5p
length: 22

mfe: -15.2 kcal/mol
p-value: undefined

position 1020
```
target 5' U  G     UGG   G  GGUGGAGC       U 3'
           GA UGUGC   AAU UG        CUGCUUC
           UU AUAUG   UUA AU        GAUGGAG
miRNA  3'    G           G                 U 5'
```

dataset: 1
TARGET : mm_Notch1_3UTR
length: 1628
MIRNA : mmu-let-7f-5p
length: 22

mfe: -14.5 kcal/mol
p-value: undefined

position 690
```
target 5'    A   GU      GGGCCUUGGGAG      U 3'
              GUA  GUUUAU            UACUUC
              UAU  UAGAUG            AUGGAG
miRNA  3' UUGA   GU                        U 5'
```

dataset: 1
TARGET : mm_Notch1_3UTR
length: 1628

MIRNA : mmu-let-7f-5p
length: 22

mfe: -9.9 kcal/mol
p-value: undefined

position 1532

```
target 5' A       GG    GA AAUGAUG      U 3'
           AACUGUA  UAAU  A       UAUUUU
           UUGAUAU  GUUA  U       AUGGAG
miRNA  3'               GA G            U 5'
```

dataset: 1
TARGET : mm_Notch1_3UTR
length: 1628
MIRNA : mmu-let-7f-5p
length: 22

mfe: -8.7 kcal/mol
p-value: undefined

position 200

```
target 5'  U                  C 3'
            UUAUAUG   UAUUGUUUU
            GAUAUGU   AUGAUGGAG
miRNA  3' UU       UAG         U 5'
```

dataset: 1
TARGET : mm_Notch1_3UTR
length: 1628
MIRNA : mmu-let-7f-5p
length: 22

mfe: -7.3 kcal/mol
p-value: undefined

position 74

```
target 5' U    GG    CCAAAGGAGCCU    AAAAA      U 3'
           GCUG  GCGA            UUUA     UGUUUU
           UGAU  UGUU            AGAU     AUGGAG
miRNA  3' U    A                     G          U 5'
```

dataset: 1
TARGET : mm_Notch1_3UTR
length: 1628
MIRNA : mmu-let-7f-5p
length: 22

mfe: 0.0 kcal/mol
p-value: undefined

position 0

```
target 5'  3'


miRNA  3'  5'
```

dataset: 1
TARGET : mm_Notch1_3UTR
length: 1628
MIRNA : mmu-let-7g-5p
length: 22

mfe: -20.7 kcal/mol
p-value: undefined

position 163

```
target 5'   A     UUUUAUUUUCCACAG             U 3'
             UGUAC               AAAC ACUGCCUU
             ACAUG               UUUG UGAUGGAG
miRNA  3' UUG                        A        U 5'
```

dataset: 1
TARGET : mm_Notch1_3UTR
length: 1628
MIRNA : mmu-let-7g-5p
length: 22

mfe: -19.6 kcal/mol
p-value: undefined

position 771

```
target 5'     A    GG UG  G      C 3'
               UGCA  G  UG UGCCUC
               AUGU  U  AU AUGGAG
miRNA  3' UUGAC       UG  G      U 5'
```

dataset: 1
TARGET : mm_Notch1_3UTR
length: 1628
MIRNA : mmu-let-7g-5p
length: 22

mfe: -19.3 kcal/mol
p-value: undefined

position 43
```
target 5'  C    UG   G CCUUUCC G       G 3'
            CUGU  GGA U       A UGCUUCA
            GACA  UUU A       U AUGGAGU
miRNA  3' UU    UG   G         G         5'
```

dataset: 1
TARGET : mm_Notch1_3UTR
length: 1628
MIRNA : mmu-let-7g-5p
length: 22

mfe: -18.5 kcal/mol
p-value: undefined

position 1005
```
target 5'    G   G    A        G 3'
              GUG GGAC   UGCCUU
              CAU UUUG   AUGGAG
miRNA  3' UUGA   G    AUG      U 5'
```

dataset: 1
TARGET : mm_Notch1_3UTR
length: 1628
MIRNA : mmu-let-7g-5p
length: 22

mfe: -18.3 kcal/mol
p-value: undefined

position 345
```
target 5' U     UU  UU    G        U 3'
           GCUGU  AU  AUU AUUGCUUC
           UGACA  UG  UGA UGAUGGAG
miRNA  3' U         UU            U 5'
```

dataset: 1
TARGET : mm_Notch1_3UTR
length: 1628
MIRNA : mmu-let-7g-5p
length: 22

mfe: -18.1 kcal/mol
p-value: undefined

position 1020
```
target 5' U  G     UGG  UG  GGUGGAGC       U 3'
           GA UGUGC   AA  UG        CUGCUUC
```

```
           UU ACAUG   UU  AU        GAUGGAG
miRNA  3'    G          UG                 U 5'
```

dataset: 1
TARGET : mm_Notch1_3UTR
length: 1628
MIRNA : mmu-let-7g-5p
length: 22

mfe: -15.0 kcal/mol
p-value: undefined

position 685

```
target 5'  U     G   UG UUAUGGGCCUUGGG G      U 3'
            CUGUA UAG  U              A UACUUC
            GACAU GUU  A              U AUGGAG
miRNA  3' UU         UG                G      U 5'
```

dataset: 1
TARGET : mm_Notch1_3UTR
length: 1628
MIRNA : mmu-let-7g-5p
length: 22

mfe: -12.1 kcal/mol
p-value: undefined

position 1532

```
target 5' A       GGUAAUGA    GAUG      U 3'
           AACUGUA        AAAU    UAUUUU
           UUGACAU        UUUG    AUGGAG
miRNA  3'         G           AUG       U 5'
```

dataset: 1
TARGET : mm_Notch1_3UTR
length: 1628
MIRNA : mmu-let-7g-5p
length: 22

mfe: -8.9 kcal/mol
p-value: undefined

position 197

```
target 5'     U   UUAU UG         C 3'
               UAU    A  UAUUGUUUU
               AUG    U  AUGAUGGAG
miRNA  3' UUGAC   U    UG         U 5'
```

dataset: 1
TARGET : mm_Notch1_3UTR
length: 1628
MIRNA : mmu-let-7g-5p
length: 22

mfe: -6.6 kcal/mol
p-value: undefined

position 74

```
target 5' U    GG    CCAAA GA  CUUUUAAAAAA      U 3'
           GCUG  GCGA     G  GC           UGUUUU
           UGAC  UGUU     U  UG           AUGGAG
miRNA  3' U    A          GA                    U 5'
```

dataset: 1
TARGET : mm_Notch1_3UTR
length: 1628
MIRNA : mmu-let-7g-5p
length: 22

mfe: 0.0 kcal/mol
p-value: undefined

position 0

```
target 5'  3'


miRNA  3'  5'
```

dataset: 1
TARGET : mm_Notch1_3UTR
length: 1628
MIRNA : mmu-let-7i-5p
length: 22

mfe: -24.2 kcal/mol
p-value: undefined

position 766

```
target 5' A   CAU   G  UG  G      C 3'
           AGC   GCA GG  UG UGCCUC
           UUG   CGU UU  AU AUGGAG
miRNA  3'     U     G  UG  G      U 5'
```

dataset: 1
TARGET : mm_Notch1_3UTR
length: 1628

MIRNA : mmu-let-7i-5p
length: 22

mfe: -21.4 kcal/mol
p-value: undefined

position 178
```
target 5'    C      AAC        U 3'
               CACAGA   ACUGCCUU
               GUGUUU   UGAUGGAG
miRNA  3' UUGUC      GA         U 5'
```

dataset: 1
TARGET : mm_Notch1_3UTR
length: 1628
MIRNA : mmu-let-7i-5p
length: 22

mfe: -19.0 kcal/mol
p-value: undefined

position 1004
```
target 5'   G    G    A        G 3'
             GGUG GGAC   UGCCUU
             UCGU UUUG   AUGGAG
miRNA  3' UUG    G    AUG      U 5'
```

dataset: 1
TARGET : mm_Notch1_3UTR
length: 1628
MIRNA : mmu-let-7i-5p
length: 22

mfe: -18.1 kcal/mol
p-value: undefined

position 1026
```
target 5'    U  UG    GUGGGUGGAGC       U 3'
              GC  GAAU           CUGCUUC
              CG  UUUG           GAUGGAG
miRNA  3' UUGU  UG    AU                U 5'
```

dataset: 1
TARGET : mm_Notch1_3UTR
length: 1628
MIRNA : mmu-let-7i-5p
length: 22

mfe: -16.6 kcal/mol
p-value: undefined

position 699
```
target 5' U UG     UU GG G      U 3'
           A  GGC C  G  A UACUUC
           U  UCG G  U  U AUGGAG
miRNA  3'   UG   U UU GA G      U 5'
```

dataset: 1
TARGET : mm_Notch1_3UTR
length: 1628
MIRNA : mmu-let-7i-5p
length: 22

mfe: -16.3 kcal/mol
p-value: undefined

position 39
```
target 5' A   CCU  UG   G CCUUUCC G       G 3'
           AGC   GU  GGA U       A UGCUUCA
           UUG   CG  UUU A       U AUGGAGU
miRNA  3'     U    UG   G         G         5'
```

dataset: 1
TARGET : mm_Notch1_3UTR
length: 1628
MIRNA : mmu-let-7i-5p
length: 22

mfe: -14.7 kcal/mol
p-value: undefined

position 345
```
target 5' U  U  UU  UU   G        U 3'
           GC GU  AU  AUU AUUGCUUC
           UG CG  UG  UGA UGAUGGAG
miRNA  3' U  U      UU            U 5'
```

dataset: 1
TARGET : mm_Notch1_3UTR
length: 1628
MIRNA : mmu-let-7i-5p
length: 22

mfe: -14.5 kcal/mol
p-value: undefined

position 150

```
target 5'  U      UU   UAUG      U 3'
            UAGUAU  AUU    UACUUU
            GUCGUG  UGA    AUGGAG
miRNA  3' UU      UU   UG        U 5'
```

dataset: 1
TARGET : mm_Notch1_3UTR
length: 1628
MIRNA : mmu-let-7i-5p
length: 22

mfe: -10.5 kcal/mol
p-value: undefined

position 1539

```
target 5'   A    AUGA    GAUG      U 3'
             GGUA    AAAU    UAUUUU
             UCGU    UUUG    AUGGAG
miRNA  3' UUG    G       AUG       U 5'
```

dataset: 1
TARGET : mm_Notch1_3UTR
length: 1628
MIRNA : mmu-let-7i-5p
length: 22

mfe: -8.6 kcal/mol
p-value: undefined

position 197

```
target 5'     U   UUAU UG         C 3'
                UAU    A  UAUUGUUUU
                GUG    U  AUGAUGGAG
miRNA  3' UUGUC   U    UG         U 5'
```

dataset: 1
TARGET : mm_Notch1_3UTR
length: 1628
MIRNA : mmu-let-7i-5p
length: 22

mfe: -7.6 kcal/mol
p-value: undefined

position 88

```
target 5' A  G   CUUU    AAA       U 3'
           AG AGC    UAAA    UGUUUU
```

```
           UU UCG    GUUU    AUGGAG
miRNA  3'    G   U       GAUG      U 5'
```

dataset: 1
TARGET : mm_Notch1_3UTR
length: 1628
MIRNA : mmu-let-7i-5p
length: 22

mfe: 0.0 kcal/mol
p-value: undefined

position 0

```
target 5'  3'


miRNA  3'  5'
```

dataset: 1
TARGET : mm_Notch1_3UTR
length: 1628
MIRNA : mmu-let-7d-5p
length: 22

mfe: -23.8 kcal/mol
p-value: undefined

position 971

```
target 5' A            CUGAUCCCCAAACUG  CUGGGUGGGGACA      G 3'
           AGCUAUGUGGCCU               GC             UGCCUU
           UUGAUACGUUGGA               UG             AUGGAG
miRNA  3'                                                  A 5'
```

dataset: 1
TARGET : mm_Notch1_3UTR
length: 1628
MIRNA : mmu-let-7d-5p
length: 22

mfe: -22.9 kcal/mol
p-value: undefined

position 766

```
target 5' A   C      GG G  G      C 3'
           AGC AUGCAG  U UG UGCCUC
           UUG UACGUU  G AU AUGGAG
miRNA  3'    A          G  G      A 5'
```

dataset: 1
TARGET : mm_Notch1_3UTR
length: 1628
MIRNA : mmu-let-7d-5p
length: 22

mfe: -20.2 kcal/mol
p-value: undefined

position 1038
```
target 5'    G   G              G 3'
              GUG AGCC   UGCUUCU
              UAC UUGG   AUGGAGA
miRNA  3' UUGA   G    AUG         5'
```

dataset: 1
TARGET : mm_Notch1_3UTR
length: 1628
MIRNA : mmu-let-7d-5p
length: 22

mfe: -19.6 kcal/mol
p-value: undefined

position 697
```
target 5'  U            UGGG G      C 3'
            UUAUG GGCCU    A UACUUCU
            GAUAC UUGGA    U AUGGAGA
miRNA  3' UU     G          G         5'
```

dataset: 1
TARGET : mm_Notch1_3UTR
length: 1628
MIRNA : mmu-let-7d-5p
length: 22

mfe: -18.6 kcal/mol
p-value: undefined

position 160
```
target 5'  U       CUUUUAUUUUCC  AGAAAC         U 3'
            UUAUGUA            AC      ACUGCCUUU
            GAUACGU            UG      UGAUGGAGA
miRNA  3' UU                     GA               5'
```

dataset: 1
TARGET : mm_Notch1_3UTR
length: 1628

MIRNA : mmu-let-7d-5p
length: 22

mfe: -17.5 kcal/mol
p-value: undefined

position 343
```
target 5'    A    UGUUU      UGAU       U 3'
              AUGC     AUUUAU    UGCUUCU
              UACG     UGGAUG    AUGGAGA
miRNA  3' UUGA    U                       5'
```

dataset: 1
TARGET : mm_Notch1_3UTR
length: 1628
MIRNA : mmu-let-7d-5p
length: 22

mfe: -15.9 kcal/mol
p-value: undefined

position 43
```
target 5'  C    U G  GU   UUCC G      A 3'
            CUGU G GA  CCU    A UGCUUC
            GAUA C UU  GGA    U AUGGAG
miRNA  3' UU      G            G      A 5'
```

dataset: 1
TARGET : mm_Notch1_3UTR
length: 1628
MIRNA : mmu-let-7d-5p
length: 22

mfe: -13.5 kcal/mol
p-value: undefined

position 74
```
target 5' U    GG      AAAGGAGCCUUU  AAAAA       A 3'
           GCUG  GCGACC            UA     UGUUUUU
           UGAU  CGUUGG            AU     AUGGAGA
miRNA  3' U    A                     G             5'
```

dataset: 1
TARGET : mm_Notch1_3UTR
length: 1628
MIRNA : mmu-let-7d-5p
length: 22

mfe: -12.0 kcal/mol
p-value: undefined

position 1520

```
target 5' A     CA    AA   GUAGGUAAUGAAAA  AUG       U 3'
           AACUA  UGCA  ACU              UG   UAUUUUU
           UUGAU  ACGU  UGG              AU   AUGGAGA
miRNA  3'                                  G           5'
```

dataset: 1
TARGET : mm_Notch1_3UTR
length: 1628
MIRNA : mmu-let-7d-5p
length: 22

mfe: -8.6 kcal/mol
p-value: undefined

position 203

```
target 5'   A                  C 3'
             UAUGU     AUUGUUUU
             AUACG     UGAUGGAG
miRNA  3' UUG     UUGGA        A 5'
```

dataset: 1
TARGET : mm_Notch1_3UTR
length: 1628
MIRNA : mmu-let-7d-5p
length: 22

mfe: 0.0 kcal/mol
p-value: undefined

position 0

```
target 5'  3'


miRNA  3'  5'
```

dataset: 1
TARGET : mm_Notch1_3UTR
length: 1628
MIRNA : mmu-let-7a-5p
length: 22

mfe: -20.7 kcal/mol
p-value: undefined

position 766

```
target 5' A   C      GG G  G      C 3'
           AGC AUGCAG  U UG UGCCUC
           UUG UAUGUU  G AU AUGGAG
miRNA  3'     A         G  G      U 5'
```

dataset: 1
TARGET : mm_Notch1_3UTR
length: 1628
MIRNA : mmu-let-7a-5p
length: 22

mfe: -18.9 kcal/mol
p-value: undefined

position 43

```
target 5'  C    UG GAGU   UUCC G       G 3'
            CUGU  G    CCU    A UGCUUCA
            GAUA  U    GGA    U AUGGAGU
miRNA  3' UU    UG U           G         5'
```

dataset: 1
TARGET : mm_Notch1_3UTR
length: 1628
MIRNA : mmu-let-7a-5p
length: 22

mfe: -18.8 kcal/mol
p-value: undefined

position 971

```
target 5' A             CUGAUCCCCAAACUG  CUGGGUGGGGACA      G 3'
           AGCUAUGUGGCCU               GC             UGCCUU
           UUGAUAUGUUGGA               UG             AUGGAG
miRNA  3'                                                   U 5'
```

dataset: 1
TARGET : mm_Notch1_3UTR
length: 1628
MIRNA : mmu-let-7a-5p
length: 22

mfe: -18.2 kcal/mol
p-value: undefined

position 179

```
target 5'      C    AAA          U 3'
                ACAG   C ACUGCCUU
```

```
                UGUU   G UGAUGGAG
miRNA  3' UUGAUA    G   A        U 5'
```

dataset: 1
TARGET : mm_Notch1_3UTR
length: 1628
MIRNA : mmu-let-7a-5p
length: 22

mfe: -16.9 kcal/mol
p-value: undefined

position 1038

```
target 5'    G   G             U 3'
              GUG AGCC   UGCUUC
              UAU UUGG   AUGGAG
miRNA  3' UUGA   G    AUG      U 5'
```

dataset: 1
TARGET : mm_Notch1_3UTR
length: 1628
MIRNA : mmu-let-7a-5p
length: 22

mfe: -16.6 kcal/mol
p-value: undefined

position 345

```
target 5' U     UU       UGAU      U 3'
           GCUGU   AUUUAU    UGCUUC
           UGAUA   UGGAUG    AUGGAG
miRNA  3' U     UGU                U 5'
```

dataset: 1
TARGET : mm_Notch1_3UTR
length: 1628
MIRNA : mmu-let-7a-5p
length: 22

mfe: -16.5 kcal/mol
p-value: undefined

position 697

```
target 5'  U           UGGG G      U 3'
            UUAUG GGCCU    A UACUUC
            GAUAU UUGGA    U AUGGAG
miRNA  3' UU     G          G      U 5'
```

dataset: 1
TARGET : mm_Notch1_3UTR
length: 1628
MIRNA : mmu-let-7a-5p
length: 22

mfe: -12.2 kcal/mol
p-value: undefined

position 152
```
target 5'    A    UU      G      U 3'
              GUAU  AUUUAU UACUUU
              UAUG  UGGAUG AUGGAG
miRNA  3' UUGA    U              U 5'
```

dataset: 1
TARGET : mm_Notch1_3UTR
length: 1628
MIRNA : mmu-let-7a-5p
length: 22

mfe: -10.9 kcal/mol
p-value: undefined

position 74
```
target 5' U    GG      AAAGGAGCCUUU  AAAAA      U 3'
           GCUG  GCGACC            UA     UGUUUU
           UGAU  UGUUGG            AU     AUGGAG
miRNA  3' U    A                     G          U 5'
```

dataset: 1
TARGET : mm_Notch1_3UTR
length: 1628
MIRNA : mmu-let-7a-5p
length: 22

mfe: -10.2 kcal/mol
p-value: undefined

position 1532
```
target 5' A       GG    GA AAUGAUG      U 3'
           AACUGUA  UAAU  A       UAUUUU
           UUGAUAU  GUUG  U       AUGGAG
miRNA  3'               GA G            U 5'
```

dataset: 1
TARGET : mm_Notch1_3UTR
length: 1628

MIRNA : mmu-let-7a-5p
length: 22

mfe: -8.8 kcal/mol
p-value: undefined

position 196

```
target 5'  U    UUAU   G         C 3'
            UUAU    AU UAUUGUUUU
            GAUA    UG AUGAUGGAG
miRNA  3' UU    UGU   G         U 5'
```

dataset: 1
TARGET : mm_Notch1_3UTR
length: 1628
MIRNA : mmu-let-7a-5p
length: 22

mfe: -6.6 kcal/mol
p-value: undefined

position 170

```
target 5'              N       C 3'
                        UUAUUUU
                        GAUGGAG
miRNA  3' UUGAUAUGUUGGAU       U 5'
```

dataset: 1
TARGET : mm_Notch1_3UTR
length: 1628
MIRNA : mmu-let-7a-5p
length: 22

mfe: 0.0 kcal/mol
p-value: undefined

position 0

```
target 5'  3'


miRNA  3'  5'
```

dataset: 1
TARGET : mm_Notch1_3UTR
length: 1628
MIRNA : mmu-let-7b-5p
length: 22

mfe: -26.2 kcal/mol
p-value: undefined

position 766
```
target 5' A          GG G  G      C 3'
           AGCCAUGCAG  U UG UGCCUC
           UUGGUGUGUU  G AU AUGGAG
miRNA  3'               G  G      U 5'
```

dataset: 1
TARGET : mm_Notch1_3UTR
length: 1628
MIRNA : mmu-let-7b-5p
length: 22

mfe: -24.1 kcal/mol
p-value: undefined

position 177
```
target 5'  U     G  AC         U 3'
            CCACA AA   ACUGCCUU
            GGUGU UU   UGAUGGAG
miRNA  3' UU     G  GGA        U 5'
```

dataset: 1
TARGET : mm_Notch1_3UTR
length: 1628
MIRNA : mmu-let-7b-5p
length: 22

mfe: -19.7 kcal/mol
p-value: undefined

position 991
```
target 5'  C   A  U     GGGUGGGG  A      G 3'
            CCA AC GGCCU        AC UGCCUU
            GGU UG UUGGA        UG AUGGAG
miRNA  3' UU   G                         U 5'
```

dataset: 1
TARGET : mm_Notch1_3UTR
length: 1628
MIRNA : mmu-let-7b-5p
length: 22

mfe: -17.2 kcal/mol
p-value: undefined

position 39

```
target 5' A    CUG UG GAGU   UUCC G       G 3'
           AGCC   U  G    CCU    A UGCUUCA
           UUGG   G  U    GGA    U AUGGAGU
miRNA  3'      U   UG U           G         5'
```

dataset: 1
TARGET : mm_Notch1_3UTR
length: 1628
MIRNA : mmu-let-7b-5p
length: 22

mfe: -16.1 kcal/mol
p-value: undefined

position 702

```
target 5'       G     UGGG G      U 3'
                  GGCCU    A UACUUC
                  UUGGA    U AUGGAG
miRNA  3' UUGGUGUG          G      U 5'
```

dataset: 1
TARGET : mm_Notch1_3UTR
length: 1628
MIRNA : mmu-let-7b-5p
length: 22

mfe: -15.9 kcal/mol
p-value: undefined

position 1022

```
target 5' A UG   UG  AUGUGGGUGGAGC       U 3'
           G  UGC  GA             CUGCUUC
           U  GUG  UU             GAUGGAG
miRNA  3'   UG   UG  GGAU                U 5'
```

dataset: 1
TARGET : mm_Notch1_3UTR
length: 1628
MIRNA : mmu-let-7b-5p
length: 22

mfe: -13.7 kcal/mol
p-value: undefined

position 330

```
target 5'  U     AUUUGUAA UG  GUUUAUUUAUUG        U 3'
            UCAUG        A  CU              AUUGCUUC
```

```
            GGUGU        U  GA             UGAUGGAG
miRNA  3' UU     G        UG                       U 5'
```

dataset: 1
TARGET : mm_Notch1_3UTR
length: 1628
MIRNA : mmu-let-7b-5p
length: 22

mfe: -13.0 kcal/mol
p-value: undefined

position 82

```
target 5' C     AA G     UUUAAAAAA      U 3'
           GACCA  G AGCCU         UGUUUU
           UUGGU  U UUGGA         AUGGAG
miRNA  3'       G  G     UG             U 5'
```

dataset: 1
TARGET : mm_Notch1_3UTR
length: 1628
MIRNA : mmu-let-7b-5p
length: 22

mfe: -12.3 kcal/mol
p-value: undefined

position 1520

```
target 5' A        GCAA    GUAGGUAAUGAAAA  AUG      U 3'
           AACUACAU    AACU              UG   UAUUUU
           UUGGUGUG    UUGG              AU   AUGGAG
miRNA  3'                                  G        U 5'
```

dataset: 1
TARGET : mm_Notch1_3UTR
length: 1628
MIRNA : mmu-let-7b-5p
length: 22

mfe: -11.7 kcal/mol
p-value: undefined

position 137

```
target 5'  U    UUUUUUUUUU GU      UUAUG      U 3'
            CCAU          A  AUUUAU     UACUUU
            GGUG          U  UGGAUG     AUGGAG
miRNA  3' UU               GU                 U 5'
```

dataset: 1
TARGET : mm_Notch1_3UTR
length: 1628
MIRNA : mmu-let-7b-5p
length: 22

mfe: -8.7 kcal/mol
p-value: undefined

position 196

```
target 5'  U    UUAU  G         C 3'
            UUAU    AU UAUUGUUUU
            GGUG    UG AUGAUGGAG
miRNA  3' UU    UGU   G         U 5'
```

dataset: 1
TARGET : mm_Notch1_3UTR
length: 1628
MIRNA : mmu-let-7b-5p
length: 22

mfe: 0.0 kcal/mol
p-value: undefined

position 0

```
target 5'  3'


miRNA  3'  5'
```

dataset: 1
TARGET : mm_Notch1_3UTR
length: 1628
MIRNA : mmu-let-7c-5p
length: 22

mfe: -27.2 kcal/mol
p-value: undefined

position 766

```
target 5' A          GG G  G      C 3'
           AGCCAUGCAG  U UG UGCCUC
           UUGGUAUGUU  G AU AUGGAG
miRNA  3'              G  G      U 5'
```

dataset: 1
TARGET : mm_Notch1_3UTR
length: 1628

MIRNA : mmu-let-7c-5p
length: 22

mfe: -19.7 kcal/mol
p-value: undefined

position 991
```
target 5'  C   A  U     GGGUGGGG  A      G 3'
            CCA AC GGCCU        AC UGCCUU
            GGU UG UUGGA        UG AUGGAG
miRNA  3' UU   A                         U 5'
```

dataset: 1
TARGET : mm_Notch1_3UTR
length: 1628
MIRNA : mmu-let-7c-5p
length: 22

mfe: -18.8 kcal/mol
p-value: undefined

position 39
```
target 5' A    CU  UG GAGU   UUCC G       G 3'
           AGCC  GU  G    CCU    A UGCUUCA
           UUGG  UA  U    GGA    U AUGGAGU
miRNA  3'         UG U           G         5'
```

dataset: 1
TARGET : mm_Notch1_3UTR
length: 1628
MIRNA : mmu-let-7c-5p
length: 22

mfe: -18.4 kcal/mol
p-value: undefined

position 177
```
target 5'  U   CAGA             U 3'
            CCA    AAC  ACUGCCUU
            GGU    UUG  UGAUGGAG
miRNA  3' UU   AUG    GA        U 5'
```

dataset: 1
TARGET : mm_Notch1_3UTR
length: 1628
MIRNA : mmu-let-7c-5p
length: 22

mfe: -16.9 kcal/mol
p-value: undefined

position 1038
```
target 5'    G   G             U 3'
              GUG AGCC   UGCUUC
              UAU UUGG   AUGGAG
miRNA  3' UUGG   G    AUG      U 5'
```

dataset: 1
TARGET : mm_Notch1_3UTR
length: 1628
MIRNA : mmu-let-7c-5p
length: 22

mfe: -16.1 kcal/mol
p-value: undefined

position 702
```
target 5'        G     UGGG G      U 3'
                  GGCCU    A UACUUC
                  UUGGA    U AUGGAG
miRNA  3' UUGGUAUG          G      U 5'
```

dataset: 1
TARGET : mm_Notch1_3UTR
length: 1628
MIRNA : mmu-let-7c-5p
length: 22

mfe: -15.3 kcal/mol
p-value: undefined

position 345
```
target 5' U     UU        UGAU      U 3'
           GCUGU   AUUUAU    UGCUUC
           UGGUA   UGGAUG    AUGGAG
miRNA  3' U     UGU                 U 5'
```

dataset: 1
TARGET : mm_Notch1_3UTR
length: 1628
MIRNA : mmu-let-7c-5p
length: 22

mfe: -12.3 kcal/mol
p-value: undefined

position 82
```
target 5' C     AA G     UUUAAAAAA      U 3'
           GACCA  G AGCCU        UGUUUU
           UUGGU  U UUGGA        AUGGAG
miRNA  3'       A  G     UG             U 5'
```

dataset: 1
TARGET : mm_Notch1_3UTR
length: 1628
MIRNA : mmu-let-7c-5p
length: 22

mfe: -12.2 kcal/mol
p-value: undefined

position 152
```
target 5'   A    UU      G      U 3'
              GUAU  AUUUAU UACUUU
              UAUG  UGGAUG AUGGAG
miRNA  3' UUGG    U              U 5'
```

dataset: 1
TARGET : mm_Notch1_3UTR
length: 1628
MIRNA : mmu-let-7c-5p
length: 22

mfe: -10.6 kcal/mol
p-value: undefined

position 1525
```
target 5'  A      AA   GUAGGUAAUGAAAA  AUG      U 3'
             CAUGCA  ACU              UG   UAUUUU
             GUAUGU  UGG              AU   AUGGAG
miRNA  3' UUG                           G        U 5'
```

dataset: 1
TARGET : mm_Notch1_3UTR
length: 1628
MIRNA : mmu-let-7c-5p
length: 22

mfe: -8.6 kcal/mol
p-value: undefined

position 201
```
target 5'    U    UG          C 3'
               UAUA  U AUUGUUUU
```

```
                AUGU   G UGAUGGAG
miRNA  3' UUGGU    UG A        U 5'
```

dataset: 1
TARGET : mm_Notch1_3UTR
length: 1628
MIRNA : mmu-let-7c-5p
length: 22

mfe: 0.0 kcal/mol
p-value: undefined

position 0

```
target 5'  3'


miRNA  3'  5'
```

dataset: 1
TARGET : mm_Notch1_3UTR
length: 1628
MIRNA : mmu-let-7e-5p
length: 22

mfe: -20.3 kcal/mol
p-value: undefined

position 43

```
target 5'  C    UG GAGU     CCAG       G 3'
            CUGU  G    CCUUU    UGCUUCA
            GAUA  U    GGAGG    AUGGAGU
miRNA  3' UU    UG U                    5'
```

dataset: 1
TARGET : mm_Notch1_3UTR
length: 1628
MIRNA : mmu-let-7e-5p
length: 22

mfe: -19.3 kcal/mol
p-value: undefined

position 1026

```
target 5' U    GA  UG GGGUGGAG        U 3'
           GCUG   A  U        CCUGCUUC
           UGAU   U  G        GGAUGGAG
miRNA  3' U    AUG UG A               U 5'
```

dataset: 1
TARGET : mm_Notch1_3UTR
length: 1628
MIRNA : mmu-let-7e-5p
length: 22

mfe: -18.7 kcal/mol
p-value: undefined

position 971
```
target 5' A              UGAUCCCCAAACUGG CUGGGUGGGGACA      G 3'
           AGCUAUGUGGCCUC               C             UGCCUU
           UUGAUAUGUUGGAG               G             AUGGAG
miRNA  3'                                                   U 5'
```

dataset: 1
TARGET : mm_Notch1_3UTR
length: 1628
MIRNA : mmu-let-7e-5p
length: 22

mfe: -17.9 kcal/mol
p-value: undefined

position 766
```
target 5' A   C      GG G GG      C 3'
           AGC AUGCAG  U U  UGCCUC
           UUG UAUGUU  A G  AUGGAG
miRNA  3'     A      GG G         U 5'
```

dataset: 1
TARGET : mm_Notch1_3UTR
length: 1628
MIRNA : mmu-let-7e-5p
length: 22

mfe: -17.3 kcal/mol
p-value: undefined

position 179
```
target 5'      C    AAACA       U 3'
                ACAG     CUGCCUU
                UGUU     GAUGGAG
miRNA  3' UUGAUA    GGAG        U 5'
```

dataset: 1
TARGET : mm_Notch1_3UTR
length: 1628

MIRNA : mmu-let-7e-5p
length: 22

mfe: -16.4 kcal/mol
p-value: undefined

position 697

```
target 5'  U            GGGAG      U 3'
            UUAUG GGCCUU     UACUUC
            GAUAU UUGGAG     AUGGAG
miRNA  3' UU     G      G          U 5'
```

dataset: 1
TARGET : mm_Notch1_3UTR
length: 1628
MIRNA : mmu-let-7e-5p
length: 22

mfe: -15.7 kcal/mol
p-value: undefined

position 345

```
target 5' U     UU     AUUGA       U 3'
           GCUGU   AUUU     UUGCUUC
           UGAUA   UGGA     GAUGGAG
miRNA  3' U     UGU    G           U 5'
```

dataset: 1
TARGET : mm_Notch1_3UTR
length: 1628
MIRNA : mmu-let-7e-5p
length: 22

mfe: -12.5 kcal/mol
p-value: undefined

position 74

```
target 5' U    GG      AAAGGAG  UUUUAAAAAA      U 3'
           GCUG  GCGACC       CC          UGUUUU
           UGAU  UGUUGG       GG          AUGGAG
miRNA  3' U    A       A                        U 5'
```

dataset: 1
TARGET : mm_Notch1_3UTR
length: 1628
MIRNA : mmu-let-7e-5p
length: 22

mfe: -12.5 kcal/mol
p-value: undefined

position 160
```
target 5'  U     UA            C 3'
            UUAUG    CUUUUAUUUU
            GAUAU    GAGGAUGGAG
miRNA  3' UU     GUUG          U 5'
```

dataset: 1
TARGET : mm_Notch1_3UTR
length: 1628
MIRNA : mmu-let-7e-5p
length: 22

mfe: -11.4 kcal/mol
p-value: undefined

position 1532
```
target 5' A       GGUAAUGAA   GA G      U 3'
           AACUGUA         AAU  U UAUUUU
           UUGAUAU         UUG  G AUGGAG
miRNA  3'         G           GA G      U 5'
```

dataset: 1
TARGET : mm_Notch1_3UTR
length: 1628
MIRNA : mmu-let-7e-5p
length: 22

mfe: -6.9 kcal/mol
p-value: undefined

position 201
```
target 5'     U    UG A        C 3'
               UAUA  U  UUGUUUU
               AUGU  G  GAUGGAG
miRNA  3' UUGAU    UG AG       U 5'
```
